# Early-Life Exposure to Environmental Contaminants Perturbs the Sperm Epigenome and Induces Negative Pregnancy Outcomes for Three Generations via the Paternal Lineage

**DOI:** 10.3390/epigenomes5020010

**Published:** 2021-05-01

**Authors:** Clotilde Maurice, Mathieu Dalvai, Romain Lambrot, Astrid Deschênes, Marie-Pier Scott-Boyer, Serge McGraw, Donovan Chan, Nancy Côté, Ayelet Ziv-Gal, Jodi A. Flaws, Arnaud Droit, Jacquetta Trasler, Sarah Kimmins, Janice L. Bailey

**Affiliations:** 1 Research Centre on Reproduction and Intergenerational Health, Department of Animal Sciences, Université Laval, Quebec City, QC G1V 0A6, Canada; clotilde.maurice@canada.ca (C.M.); mathieu.dalvai@fsaa.ulaval.ca (M.D.); 2 Department of Animal Sciences, McGill University, Ste. Anne de Bellevue, Quebec, QC H9X 3V9, Canada; romain.lambrot@mcgill.ca (R.L.); sarah.kimmins@mcgill.ca (S.K.); 3 Department of Pharmacology and Therapeutics, McGill University, Montreal, QC H3G 1Y6, Canada; 4 Department of Molecular Medicine, Research Center of CHU of Quebec City, Université Laval, Quebec City, QC G1V 4G, Canada; deschene@cshl.edu (A.D.); MariePier.ScottBoyer@crchudequebec.ulaval.ca (M.-P.S.-B.); arnaud.droit@crchudequebec.ulaval.ca (A.D.); 5 Research Center of CHU Sainte-Justine, Department of Biochemistry and Molecular Medicine, Université de Montral, Montreal, QC H3T 1C5, Canada; serge.mcgraw@umontreal.ca; 6 Research Institute of the McGill University Health Centre, Montreal, QC H3Z 2Z3, Canada; donovan.chan@mail.mcgill.ca (D.C.); jacquetta.trasler@mcgill.ca (J.T.); 7 Departments of Pediatrics, Human Genetics and Pharmacology & Therapeutics, McGill University, Montreal, QC H3Z 2Z3, Canada; 8 Institut Universitaire de Cardiologie et de Pneumologie de Québec, Université Laval, Quebec City, QC G1V 4G5, Canada; nancy.cote@criucpq.ulaval.ca; 9 Department of Comparative Biosciences, University of Illinois, Urbana-Champaign, IL 61802, USA; zivgal1@illinois.edu (A.Z.-G.); jflaws@illinois.edu (J.A.F.)

**Keywords:** persistent organic pollutants (POPs), sperm methylome, intergenerational, transgenerational, epigenetic, paternal transmission, Inuit

## Abstract

Due to the grasshopper effect, the Arctic food chain in Canada is contaminated with persistent organic pollutants (POPs) of industrial origin, including polychlorinated biphenyls and organochlorine pesticides. Exposure to POPs may be a contributor to the greater incidence of poor fetal growth, placental abnormalities, stillbirths, congenital defects and shortened lifespan in the Inuit population compared to non-Aboriginal Canadians. Although maternal exposure to POPs is well established to harm pregnancy outcomes, paternal transmission of the effects of POPs is a possibility that has not been well investigated. We used a rat model to test the hypothesis that exposure to POPs during gestation and suckling leads to developmental defects that are transmitted to subsequent generations via the male lineage. Indeed, developmental exposure to an environmentally relevant Arctic POPs mixture impaired sperm quality and pregnancy outcomes across two subsequent, unexposed generations and altered sperm DNA methylation, some of which are also observed for two additional generations. Genes corresponding to the altered sperm methylome correspond to health problems encountered in the Inuit population. These findings demonstrate that the paternal methylome is sensitive to the environment and that some perturbations persist for at least two subsequent generations. In conclusion, although many factors influence health, paternal exposure to contaminants plays a heretofore-underappreciated role with sperm DNA methylation contributing to the molecular underpinnings involved.

## 1. Introduction

The World Health Organization states that direct exposure to persistent organic pollutants (POPs), even at low doses, can lead to an increased risk of cancer, reproductive disorders, altered immune response, neuro-behavioural impairment and endocrine disruption [1,2]. Although the Stockholm Convention restricts the production and use of POPs, the organochlorine pesticide, dichlorodiphenyltrichloroethane (DDT), is still used to combat malaria-transmitting mosquitos [3].

Although never used in the Arctic, traditional foods of northern Aboriginal populations are persistently contaminated with POPs because of global weather currents [4,5,6]. Climate change and the release of POPs from melting ice appear to be exacerbating the problem [7,8].

There are major health disparities between Inuit and non-Aboriginal Canadians such that the life expectancy is 14 years shorter among Inuit [9,10]. The risks of neonatal and postnatal infant death are substantially elevated in Inuit communities [11]. Stillbirths are 1.9 times more frequent among Inuit than non-Aboriginal residents in the Canadian province of Quebec and are attributed to poor fetal growth, placental and congenital disorders [12]. A study of northern Quebec Inuit communities revealed that prenatal exposure to POPs is correlated with shorter pregnancy and with lower infant birth weight, length and head circumference [12]. Lower alertness in Inuit infants [13], more frequent respiratory infections [14], and slower emotional development [15] in Inuit preschoolers have also been correlated with in utero exposure to PCBs. 

While it is established that maternal environment (e.g., diet, POPs exposure) can affect child health and development, recent research indicates that negative effects can be transmitted to future generations via the fathers. Epidemiological data indicate that paternal lifestyle is associated with disease occurring in at least two subsequent generations [16,17,18,19]. Animal studies have shown that the gestational environment of a male is associated with adverse health outcomes in its progeny [20,21] and multiple generations of descendants, which are associated with perturbation of the paternal or ancestral sperm epigenome [22,23,24]. 

POPs have been related to the development of chronic diseases. Multiple epidemiological studies in different European countries highlighted the strong correlation between the presence of POPs in the circulating system, and the high risk of metabolic and cardiovascular diseases [25,26,27,28,29]. Recently, non-diabetic and diabetic mouse models showed that POPs exposure promotes metabolic syndrome independently of the diet by intensify oxidative and inflammatory stressors, which has for consequence to exacerbate the adverse cardiac remodeling [30]. In an epigenetic transgenerational inheritance rat model, an in utero exposure of a POP pesticide identified sperm differential DNA methylation regions that were associated with reproductive adverse effects but also with obesity across three male generations [31]. These studies suggest that POPs may also induce metabolic disorders by changing epigenetic patterns. Other study presented inherited DNA methylation modifications, non-coding RNA modifications and histone retention across generations) in sperm after an in utero exposure of POPs confirming their impact on epigenome and the risk for the next generation to inherit adverse outcomes from their father [31]. 

Given that the paternal impact of environmentally relevant contaminants is relatively unexplored, we used an established rat model [32,33] to test the hypothesis that sperm from males exposed in utero and via suckling to a POPs mixture, similar to that found in the Inuit diet, undergo epigenome modifications that have heritable developmental consequences. Here, we show that POPs exposure modified the sperm DNA methylome in a manner that is partly transmitted over generations via the paternal line independent of maternal effects. We also show reduced sperm quality across two generations and perturbed methylation patterns in sperm DNA in the third generation. These results suggest a mechanistic basis for the significant health disparities between Inuit and non-Aboriginal populations, and is the first to demonstrate that environmental exposure to Arctic contaminants can modify the male epigenome and thereby affect the health of the descendants.

## 2. Materials and Methods

### 2.1. POPs Mixture and Animals

**Figure 1:**
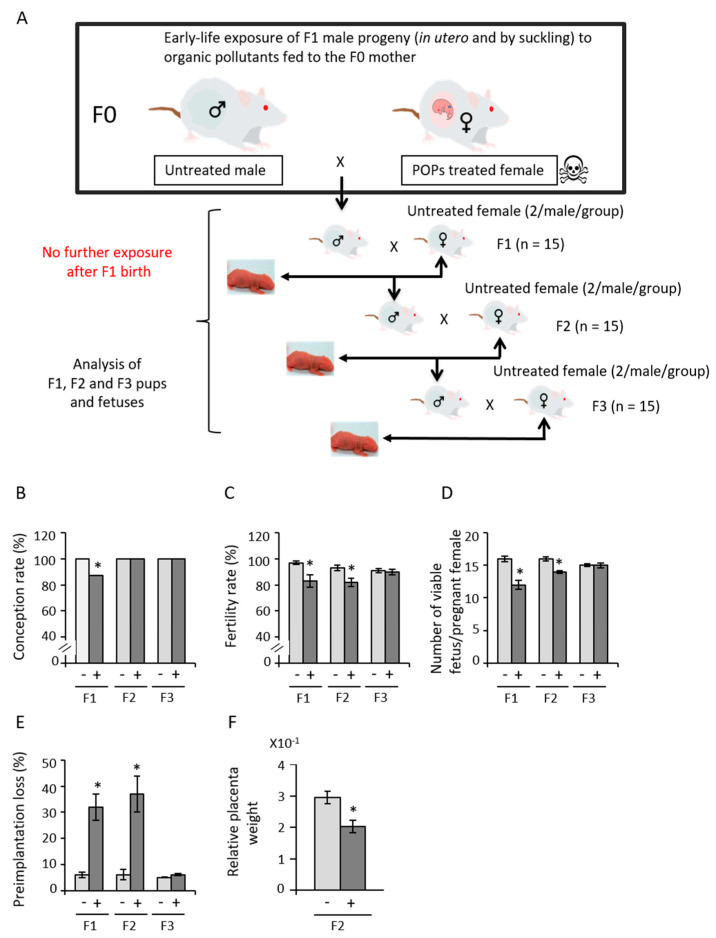
Adverseeffects induced by paternal early-life exposure to POPs on fertility parameters through three male generations (**A**) Mating and exposure scheme; (**B**) Ratio of males that conceived pups by mating with untreated females calculated as number of pregnant females/number of total females * 100; (**C**)Fertility rate for males from each generation in each group calculated as number of viable fetuses at GD 19.5/number of *corpora lutea* * 100; (**D**) Ratio of viable fetuses per pregnant femaleat GD 19.5 calculated as the average of number viable fetuses per pregnant female per group; (**E**) Ratio of unfertilized or non-implanted embryos calculated as [number of *corpora lutea—* number of implantation sites]/number of *corpora lutea* * 100 at GD19.5; (**F**) Relative placenta weight defined by the placenta weight/fetus weight at GD19.5. “−” = control group, “+” = POPs-exposed group. Significant differences due to early life treatment are based on one-way analysis of variance (ANOVA) followed by unpaired Student *t*-test. Values are presented as average ± SEM for B to E * *p* < 0.05 (*n =* 30 females/group for (**B**); *n =* 25 pregnant females at GD 19.5/group for (**C**–**E**).

The compounds were dissolved in corn oil (Sigma-Aldrich, Oakville, ON, Canada) to obtain a stock solution containing 5 mg POPs/mL, which was kept shielded from light at room temperature and diluted with corn oil to 500 µg/kg body weight/mL for administration by gavage (Table S1). Eight five-week-old females (F0 founders dams) and four ten-week-old males (untreated F0 sires) Sprague Dawley rats (Charles River Laboratories, St. Constant, QC, Canada) were allowed to acclimatize for 10 days prior to experimentation. Housing conditions were as follows: photoperiod 12L/12D, temperature 22 ± 1 °C, humidity 46 ± 10%, and water and standard commercial rat chow provided ad libitum. The experimental design is summarized in Figure 1A. F0 females assigned randomly to two groups (*n =* 8; 2 per cage) received corn oil (1 mL/100 g body weight) containing 0 µg (control) or 500 µg POPs mixture per kg of body weight three times a week for 5 weeks before mating then through mating and parturition of the F1 litters. The body weight of each female was assessed before each gavage to determine the quantity of POPs to be administered. Each pair of females was housed with an unexposed F0 sire until mating confirmation by the presence of sperm vaginal smears. Four F0 pregnant females per group were kept to produce the F1 pups and four F0 pregnant females were anaesthetized with isofurane (Baxter, Mississauga, ON, Canada), euthanized by cardiac puncture following by asphyxia with CO_2_ and caesarean-sectioned at gestational day (GD) 19.5 to analyze the F1 fetuses. 

### 2.2. F1, F2 and F3 Progeny Outcome and Male Fertility

At 90 days of age, F1, F2 and F3 males were selected randomly from each group (*n =* 15, minimum of 3 from 4 litters) and crossed with 10-week-old unexposed virgin females purchased as required from Charles River Canada Ltd. (2 females/male/generation). Each male was housed overnight with 2 females during the night only for a maximum of seven consecutive nights until mating was confirmed the next morning by the presence of sperm in vaginal smears. The conception rate was defined by the ratio of males that induced pregnancy by mating with untreated females calculated as number of pregnant females/number of total females * 100.

Twenty-five sperm-positive females per group and generation were anaesthetized with isofurane, euthanized by cardiac puncture following by asphyxia with CO_2_ and caesarean-sectioned at GD19.5 after mating to assess fertility rate defined as number of viable fetuses at GD 19.5/number of corpora lutea*00, number of viable fetus per pregnant female, pre-implantation loss defined as the ratio of unfertilized or non-implanted embryos ([number of corpora lutea—number of implantation sites]/number of corpora lutea*100), and the relative placenta weight. 

Five sperm-positive females per group and generation (mated with a minimum of 4 non-relative males) were kept to give birth to the next generation. Assessment of litter size, sex ratio, live birth ratio, pup body weight and anogenital distance were adapted from methods published previously [34]. F1, F2 and F3 litters were adjusted to the greatest number of males in POPs-exposed litters. Litters with fewer males were cross-fostered with female pups from litters in the same exposure group. Pups remained with their birth dams or foster mother until weaning at postnatal day (PND) 21. Neonatal mortality was calculated as the difference between the number of implantation sites in the uterus of sacrificed dam as previously described and the number of total pups in the corresponding litter.

Male rats were anesthetized by isofurane and euthanized by cardiac puncture following by asphyxia with CO_2_ the week after mating. Body weights were recorded. Reproductive organs were collected, weighted then snapped freeze in nitrogen and stored at −80 °C. 

### 2.3. Male Reproductive Assessment for F1, F2, F3

#### 2.3.1. Sperm Analysis

During male necropsies, the left caudal epididymis was excised, trimmed of fat, sliced and placed in a 35 mm Petri dish containing phosphate buffered saline (PBS; 1.5 mM Kh_2_PO_4_, 8.1 mM Na_2_HPO_4_, 137 mM NaCl, 2.7 mM KCl; pH 7.4) (Sigma-Aldrich, Oakville, ON, Canada) at 37 °C for 15 min in a humidified 5% CO_2_ incubator to release the fresh sperm. Sperm was washed two times with hypotonic buffer (0.45% NaCl) (Sigma-Aldrich, Oakville, ON, Canada) to lyse any contaminating cells then washed 2 times with PBS, and snapped freeze in nitrogen in 1 ml of PBS then stored at −80 °C to perform the caudal Sperm Chromosomal Protein Composition and the Sperm Chromatin Structure Assay later. The caudal epidydmal sperm cell count was done using a hemacytometer before to freeze the sample. 

The right caudal epididymis was excised, trimmed of fat, sliced and placed in another 35 mm Petri dish containing 5 ml Gibco^®^Media-199 without phenol red (Life Technologies, Grand Island, NY, USA) with 0.5% fatty acid free BSA (Sigma-Aldrich, Oakville, ON, Canada) at 37 °C for 15 min in a humidified 5% CO_2_ incubator to perform the Sperm Motion Parameters, Viability and Acrosomal Integrity, and Capacitation Assay the same day of necropsy. After the analyses, the rest of the sperm was washed two times with hypotonic buffer (0.45% NaCl) to lyse any contaminating cells then strained through a BD Falcon 70-µm nylon cell stainer (VWR International, Missisauga, ON, Canada) and washed 2 times with PBS. Spermatozoa were counted on a hematocyter and visually inspected for somatic contamination. No somatic cells were detected. Total cell suspension was centrifuged at 1000× *g* for 10 min at 4 °C then stored at −80 °C to perform the Reduced representation bisulphite sequencing (RRBS).

*Sperm motion parameters:* Using a Hamilton-Thorne CEROS II Analyzer (version 14; Beverly, MA, USA) with a 4X objective and a 100 µm deep Rat Toxicology Slide (Leja 2, Leja Company, Nieuw-Vennep, The Netherlands), the percentage of motile and progressively motile sperm, average path velocity (VAP), straight-line velocity (VSL), curvilinear velocity, amplitude of lateral head displacement (ALH), beat cross frequency (BCF), straightness and linearity were measured with a fresh sperm aliquots containing 2 × 10^6^ sperm/mL diluted 1/10 in pre-warmed PBS at 37 °C. CEROS settings were: frame rate 60 Hz; frames acquired 30; minimal contrast 80; minimal cell size 8; cell size 25; cell intensity 80; path velocity 50 µ/s; straightness 25%; slow cell motility; VAP cut-off 10 µ/s; VSL cut-off 10 µ/s; intensity 2300; photometer 2; magnification 0.7 [33].

*Sperm viability and acrosomal integrity:* As described in Maurice et al., 2018 [33], fresh sperm aliquot (2 × 10^6^/mL) was diluted in pre-warmed PBS then treated with 48 µM PI 1 µg/mL of FITC-labeled peanut-agglutinin (FIFTC-PNA; fluoroisothianocynate-labeled *Arachis hypogaea* lectin) ( Life Technologies, Grand Island, NY, USA) and incubated for 10 min at 37 °C in the dark. A total of 10,000 sperm per sample were analysed using a Guava EasyCyte Plus flow cytometer with Cytosoft software (Guava Technologies/IMV Technologies, L’Aigle, France). Results are presented as percentages of “live sperm”, “sperm with intact acrosomes” and “live sperm with intact acrosomes”.

*Capacitation assay*: The chlortetracycline fluorescence (CTC) assay was used to evaluate sperm physiological status (capacitation, spontaneous acrosome reaction) [33]. In brief, the CTC stock solution contained 750 μM (final concentration) CTC–HCl, 130 mM NaCl, 5 mM L-cycteine and 20 mM Tris acid (pH 7.8) (Sigma-Aldrich, Oakville, ON, Canada) was prepared freshly and shielded from light at 10 °C before using. A total of 10 μL of spermatozoal suspension from the previous fresh sperm aliquot containing 2 × 10^6^/mL sperm in PBS were mixed 10 μL CTC stock solution, 2 μL of 12.5% glutaraldehyde (Sigma-Aldrich, Oakville, ON, Canada) in 20 mM Tris—HCl (pH 7.4) (Sigma-Aldrich, Oakville, ON, Canada) and 25 μL of 1.4-diaza-byciclo (2.2.2) octane (0.22 M) (Sigma-Aldrich, Oakville, ON, Canada) on a clean slide at room temperature. Finally, a drop of glycerol was added to retard the fading of CTC fluorescence. The sample (two replicates per slide) were covered with coverslips and stored in the dark at 4 °C overnight. For evaluation of the CTC patterns, the slides were observed within 24 h under an Optiphot-2 microscope (Nikon Canada Inc., Mississauga, ON, Canada) equipped with phase contrast and epifluorescence optics with a 10× ocular and 40× objective using a BV filter. At least, 200 spermatozoa per sample were classified according to one of three CTC staining patterns as described in Maurice et al., 2018 [33]: F pattern = uncapacited cell with uniform bright fluorescence over the head; B pattern = intermediate pattern with a dark band (arrow) in the postacrosomal region of the sperm head and AR pattern = acrosome-reacted cell with dark head except for the tip, which retained some fluorescence.

*Sperm chromosomal protein composition*: A slide-based chromomycin A3 (CMA3) staining assay was performed to evaluate the relative abundance of protamine in sperm chromatin, based on the principle that CMA3 does not reach DNA in the presence of protamine [35]. Briefly, air-dried sperm smears from previous the fresh sperm aliquot containing 2 × 10^6^/mL sperm in PBS, were fixed in methanol/acetic acid (3:1) (Sigma-Aldrich, Oakville, ON, Canada) for 10 min at 4 °C. Each slide was covered for 20 min with 100 µL CMA3 solution (0.25 mg/mL in McIlvaine buffer [17 mL of 0.1 mol/L citric acid mixed with 83 mL of 0.2 mol/L Na_2_ HPO_4_ and 10 mmol/L MgCl_2_, pH 7.0]) (Sigma-Aldrich, Oakville, ON, Canada) at room temperature in the dark. Slides were rinsed in PBS, air-dried, mounted with glycerol and viewed an Optiphot-2 microscope (Nikon Canada Inc.) equipped with phase contrast and epifluorescence optics with a 10× ocular and 40× objective using a B2A filter (excitation at 400–440 nm and emission at 470 nm). All slides were prepared in duplicate and a total of 200 spermatozoa per slide were scored (400X). Highly fluorescent green stained spermatozoa were considered CMA3+ (positive staining, without protamine) and weakly fluorescent cells were considered CMA3- (negative staining, containing protamine). 

*Sperm chromatin stability assay*: Acridine orange (AO) staining was combined with flow cytometry for the rat sperm chromatin structure assay (SCSA^®^) as described by Maurice et al., 2018 [36] with some modifications. In brief, sperm sample from the frozen sperm aliquot was thawed for 2 min at 37 °C then diluted in a Tris Sodium Ethylenediaminetetracetic acid (TNE) buffer (0.15 M NaCl, 0.001 M EDTA, 0.01 M Tris-HCl, pH 7.4; Sigma-Aldrich, Oakville, ON, Canada) to 4 × 10^6^ sperm/mL in 200 μL to be sonicated on ice to isolate the rat sperm head from their tails. To denature uncondensed sperm DNA, sperm diluted in TNE were mixed with 400 µL of acid-detergent solution (0.08 N HCl, 0.15 M NaCl, and 0.1% Triton X-100, pH 1.4; Sigma-Aldrich, Oakville, ON, Canada) for 30 s at 4 °C then mixed with 1.2 mL of AO staining solution (0.126 M Na_2_HPO_4_, 0.037 M citric acid buffer, 1 mM EDTA, 0.15 M NaCl, pH 6.0) containing 6 µg/mL AO (EMD Millipore, Billerica, MA, USA) and incubate for 2 min and 30 s. Sperm were analysed using a using a Guava EasyCyte Plus flow cytometer and the data were processed using EasySoft CompDNA software (Guava Technologies/IMV Technologies, L’Aigle, France). The AO parameters obtained from this analysis included DNA fragmentation index (DFI = mean red fluorescence/total red + green fluorescence), which is the percentage of cells outside the main population (% DFI). At least 10,000 sperm were analyzed for each sample.

#### 2.3.2. Daily Testicular Spermatid Production

Daily testicular spermatid production was measured as described previously in Maurice et al. 2018 [33]. Briefly, the thawed testis without the tunica, one for each mated male, was homogenized in 10% DMSO/0.9% NaCl (Sigma-Aldrich, Oakville, ON, Canada) using a Polytron VDI 12 (VWR International, Radnor, PA, USA) twice for 15 s with a 30 s pause between. Samples were sonicated at 40% power for 1 min and 0.1% trypan blue (Sigma-Aldrich, Oakville, ON, Canada) was added to colour the spermatid heads, which were counted using a hemacytometer. Daily testicular spermatid production was calculated according to the equation: ((Mean count of hemacytometer/0.00004 μL volume of secondary square in hemacytometer) × 100.5 mL of total number of rat testis suspension)/6.10 days for spermatogenesis cycle.

#### 2.3.3. Histological Evaluation of Follicle Numbers

Ovaries collected from the F0 founder females (*n =* 4) and their F1 offspring (*n =* 11) from control and POPs groups were fixed in Bouin solution and paraffin, serially sectioned (8 μm) and stained with hematoxylin and eosin. Every 20th section was counted and the total number of primordial, primary, preantral, and antral follicles per ovary was calculated according to methods published previously [37,38]. These data were used to calculate the percent of each follicle type per ovary. Sections were counted on a double-blind basis.

#### 2.3.4. Testosterone Assay

After cardiac puncture, serum was obtained by centrifuging the blood of each male at 1500 g for 20 min at 4 °C then stored at −80 °C as previously published by Maurice et al., 2018 [33]. Total serum testosterone concentrations were determined in 50 µL of sample using the Testosterone EIA kit (Cayman Chemical, Ann Arbor, MI, USA) following manufacturer’s conditions with the suggested intra-assay and inter-assay coefficients of variation (CV = standard deviation/mean × 100, <15%). The IC50 and the detection limit of the assay were, respectively, 32 pg/mL and 6 pg/mL. Samples from all groups were analyzed on the same day.

#### 2.3.5. Sperm DNA Methylation Assays

*Reduced representation bisulphite sequencing (RRBS):* A gel-free technique requiring 500 ng of DNA was used according to protocols published previously [39,40]. The frozen sperm from the right epididymis was used. The 12 samples of sperm (1 sample per animal) per treatment and per generation were multiplexed in paired-end sequencing in 1 lane of a HiSeq 2000 sequencer (Illumina, San Diego, CA). Reads were trimmed using Trim Galore v0.4.0 (http://www.bioinformatics.babraham.ac.uk/projects/trim_galore; Accessed on 1st March 2021) and aligned on the Rattus norvegicus bisulphite-sequenced genome (Rnor_5.0) using bismark [41]. The methylKit package (version 0.9.4) was used for data processing and analysis [42] (100 bp tiling windows, 1 CpG minimum per tile, discarding of bases having coverage at > the 99.9th percentile,). A high stringent cut-off for methylation site validation was applied with 10 reads per sample, a q value <= 0.01 and ±≥20% average difference between groups of replicates (COV10_Met20). DMS between cases and controls is calculated for each generation. 

Differentially methylated sequences were annotated using HOMER annotatePeaks version 4.7 [43]. A homemade R script was developed to convert gene identifiers from rat to human (the script changed the definition of the HOMER TSS category called promoter to fit −2 kb to +100 bp). The script used the biomaRt package [44] to search for GRCh37 equivalents of rat EntrezGene identifiers. A final list of GRCh37 EntrezGene identifiers was used in subsequent gene enrichment analysis. DAVID (Database for Annotation Visualization and Integrated Discovery) v6.8 software was used to identify enriched functionally related gene groups and calculate an enrichment *p* value for each pathway term. Pathways were identified as significant at *p* < 0.05. Due to the incompleteness of the rat sperm genome intergenic region information (CTCF, insulator…) and since HOMER assigned the closest gene to the methylated sites identified in an intergenic region, we excluded from the bio-informatics analysis any gene assigned for a methylated site found in an intergenic region. 

The potential effects of POPs across multiple generation on biological process and pathologies were analysed by performing a functional analysis using DAVID 6,8 software according to Gene Ontology (GO) using Rattus norvegicus genome *q* value ≤ 0.01 [45]. After the conversion of identifiants (ID) from rat to human using BioDBnet software [46], pathologies associated with deregulated genes were identified.

*DNA pyrosequencing:* The degree of sperm DNA methylation was validated by pyrosequencing using a PyroMark Q24 (Qiagen, Mississauga, ON, Canada). A DNeasy Mini Kit (Qiagen, Mississauga, ON, Canada) was used to extract DNA, of which 1 µg was processed with an EpiTect Fast DNA bisulphite kit (Qiagen, Mississauga, ON, Canada). Bisulphite-treated DNA was eluted in 15 µL, of which 3 µL were used for PCR (HotStar DNA Polymerase, Qiagen, Mississauga, ON, Canada) with one biotinylated primer and then sequenced following the Qiagen protocol. The methylation status of each locus was analyzed individually as a T/C single nucleotide polymorphism using PolyMark Q24 advanced software (Qiagen, Mississauga, ON, Canada). 

### 2.4. Statistical Analyses

Values presented are means ±SEM. All data were analyzed using SPSS version 22.0 (SPSS Inc., Chicago, IL, USA). Significant differences due to exposure of the paternal lineage (Control versus POPs) were tested using one-way analysis of variance (ANOVA) followed by an unpaired Student *t*-test. Malformation frequency was analyzed using Fisher’s F test. Pyro-sequencing was analyzed using an unpaired Student *t*-test. For follicle counts, the Shapiro–Wilk test was used to confirm normal distribution of the data, followed by the Student *t*-test. Effects on serum testosterone were revealed using paired Student *t*-tests for all single comparisons with commercial software JMP 10. Differences were considered significant if *p* ≤ 0.05.

*Permutation analysis:* As described in Belleau et al. 2018 [47], the relationship between exposure to POPs and the number differentially methylated sites conserved over more than one generation was inferred from analysis of 3962 permutations between all cases, controls and generations. For each permutation, sites were detected using a procedure identical to the one used for the actual data. The number of sites conserved between generations F1, F2 and F3 was recalculated for each comparison. The proportions of values found at least as extreme as in the dataset were 0.00177 for hyper-methylated (56 conserved) and 0.000757 for hypo-methylated (58 conserved) sites. Finally, the convergence of the permutation analysis was validated [47].

## 3. Results

### 3.1. Early Exposure to POPs Produces Heritable Developmental Abnormalities

The POPs mixture was designed to represent the organic pollutant content of ringed seal blubber, a traditional Inuit food [32]. The 500 μg/kg/day dosage was confirmed in a previous study [32] to be environmentally relevant, because the serum contaminant levels in the F0 dams and F1 pups approximate serum levels in Inuit people (Table S1). 

The fertility and health of the F0 founders as well as their ovaries and F1 female ovaries were assessed to evaluate the effect of the POPs exposure on the female line. An analyzed of the development was performed on the fetuses to detect heritable abnormalities. The mixture of POPs organic compounds had no significant effect on F0 founder female body weight and weight gain during pregnancy [32,33]. The fertility rate, gestational duration and sex ratio at birth were also unaffected (Table S2), confirming no acute systemic toxicity due to the direct administration of these compounds at this dosage [32,33]. No differences in the numbers of ovarian follicles at different stages were observed in F0 founders and their F1 female offspring (Table S3). The next results are only on male lineage and the experimental design is summarized in Figure 1A. The effects on fertility and pregnancy noted across three generation are shown in Figure 1. Only some effects in F1 and transmitted to F2 can be observed. A decrease in conception (13%) was noted in F1 and in fertility in F1 (17%) and F2 (18%) in association with F1 early life exposure to POPs (Figure 1B,C). Similarly, the number of fetuses per litter also dropped in F1 and F2 (Figure 1D). The pre-implantation loss jumped to 32% in F1 and to 37% in F2 in association with early life exposure to POPs (Figure 1E). Sperm from POPs-exposed F1 males and their sons F2 thus fertilized fewer oocytes and/or fewer early embryos were able to implant or degenerate and resorb after implantation. In contrast, all of these fertility parameters appeared to be restored in F3. These results show that the POPs-associated sub-fertility of F1 and F2 males does not persist. In addition, although fetus and placenta appearance was normal in F1 and F3 (data not showed), the F2 relative placenta weight was reduced in association POPs-exposed F1to POPs (Figure 1F). Collectively, these observations show paternally mediated transmission of deleterious physiological effects occurring in association with early life exposure to POPs. 

**Figure 2:**
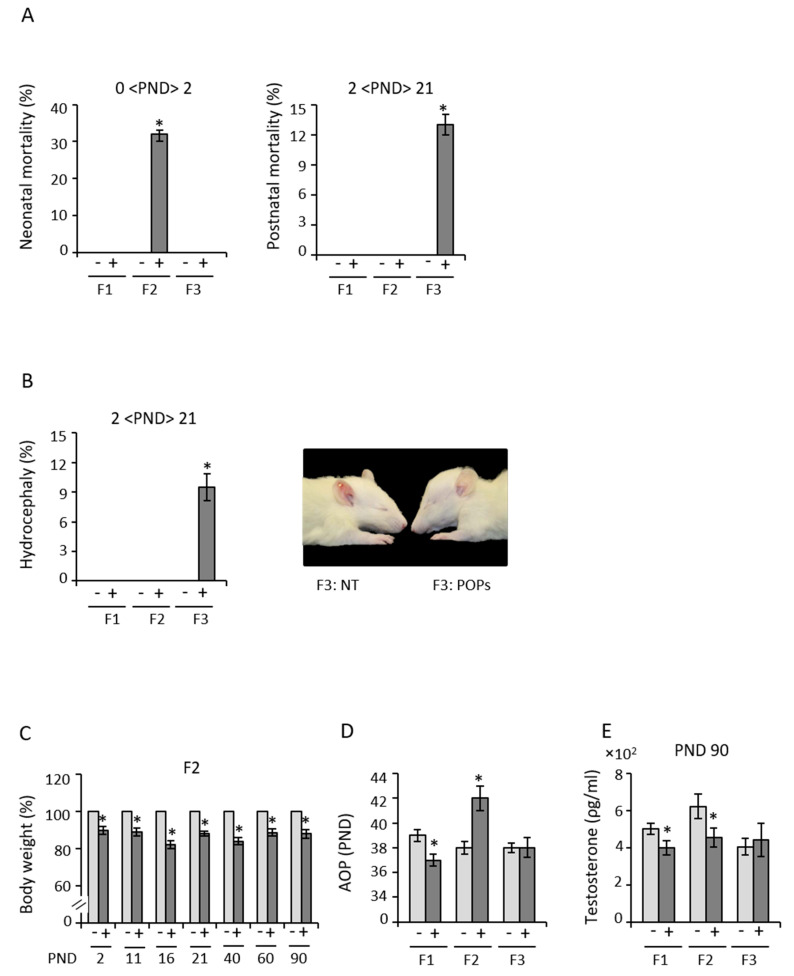
Paternal exposure to POPs induces neonatal and postnatal mortality, and birth defects. (**A**)Male and female neonatal mortality (100 − 100 * [live births/implantation sites]) between the time of birth and PND 2(30 pups from 5 different litters per treatment; 35 pups from 58 implantations in F2), (**B**) Male and female postnatal mortality (100 *[dead/total]) from PND 2 to PND 21 (time of weaning)(8 of 64 F3 pups died); (**C**) Hydrocephalus rate detected (100 * [ number of hydrocephalus/total number of pups]) in males and females before weaning (30 pups from 5 different litters per treatment, 60 pups for F1 and F3), in 6 litters of 64 F3 pups, (**D**) Picture of a normal pup from a F3 Control litter (on the **left**) and a hydrocephalus pup from a F3 POPs litter (on the **right**), (**E**) Body weight rate of F2 males from POPs exposure(+) lineage relative to control(- lineage from PND 2 to PND 90 (*n =* 5 litters of 30 rats, 6 males per litter), (**F**) Age at onset of puberty in males in each generation (*n =* 5 litters with 30 pups, puberty defined as prepuce separation), (**E**) Serum testosterone concentration at PND 90 (F1 Control *n =* 12, POPs *n =* 14, F2 Control *n =* 10, F2 POPs *n =* 11, F3 Control *n =* 7, F3 POPs *n =* 10). No differences in sex ratio, anogenital distance or testicular descent were observed between control and POPs lineages. Significant differences between linages are based on one-way analysis of variance (ANOVA) followed by unpaired Student *t*-test, values are presented as average ± *s.e.m* * *p* < 0.05.

Some of the F2 pups (32%) associated with ancestral paternal POPs exposure died within a day after birth (Figure 2A). A smaller number of F3 pups (13%) of the same lineage died within 21 days (PND 21) (Figure 2A). Of these, 9.5% (males and females) were hydrocephalic (Figure 2B), which was not observed in any other generation, regardless of paternal lineage. F2 and F3 birth morbidities and F3 congenital abnormalities were also higher in association with ancestral paternal POPs exposure. F2 males of the POPs lineage were consistently smaller from PND 2 to PND 90 (Figure 2C), whereas body weight was unaffected at this point in for all other generations (Figure S1A). However, F1 body weights then decreased until they were only 70% of the control group at one year of age and suffered a loss of fertility and health issues [33] (Figure S1B). This observation suggests that effects of POPs may be long-term, possibly across multiple generations.

In the POPs-exposed lineage, male puberty was advanced in F1, delayed considerably in F2 (Figure 2D) and then approximated the control group in F3. Paternal exposure to POPs was associated with lower plasma testosterone concentrations in F1 and F2 but not F3 (Figure 2E). However, it is also possible that some unknown factor raised F2 testosterone in the control lineage (Figure 2E). These results further show paternally transmitted intergenerational effects of POPs on the reproductive phenotype in male rats.

### 3.2. Paternal Transmission of POPs-Associated Anomalies in Reproductive Tract Development and Sperm Parameters

**Figure 3:**
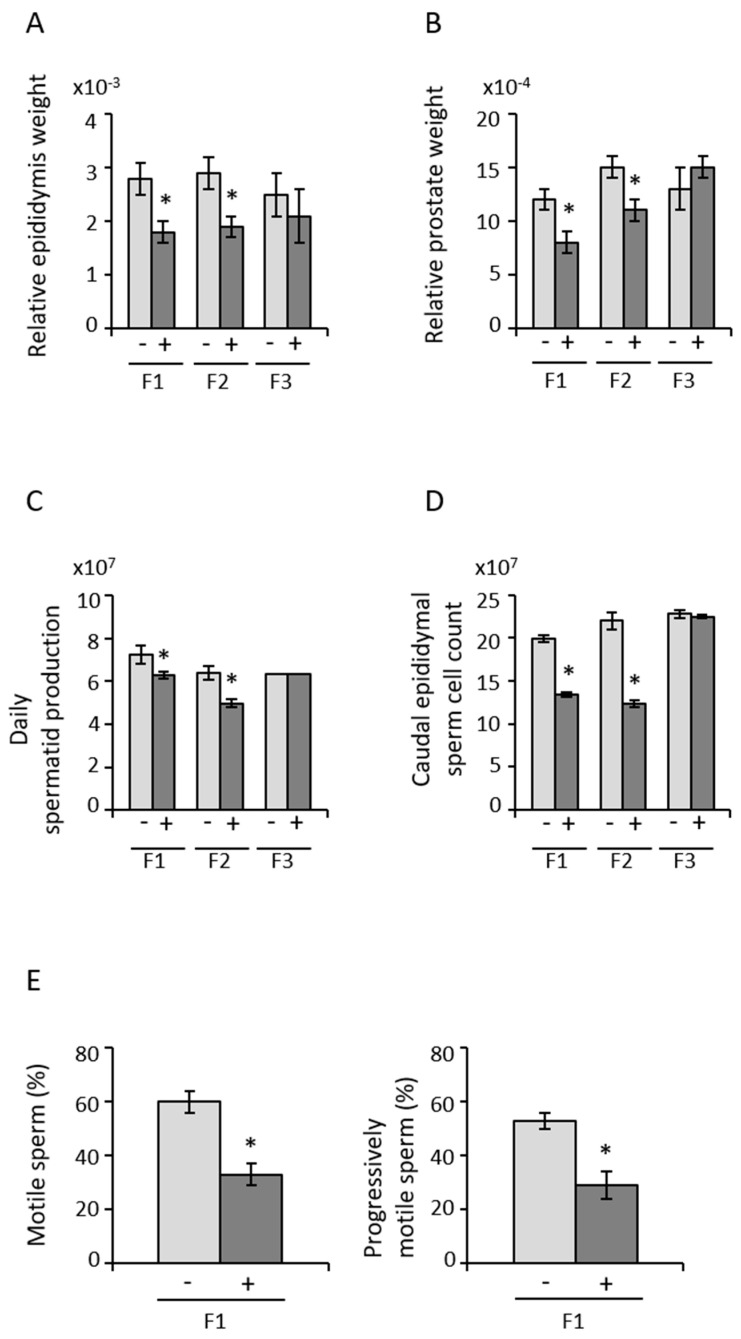
Early life exposure to POPs mixture alters reproductive tract development and modifies spermatic parameters in multiple male generations. Epididymis (**A**) and prostate (**B**) weight relative to body weight, daily spermatid production (**C**), and caudal epididymal sperm cell count (**D**); sperm cell motility parameters on day 90 after 15 min at 37 °C (**E**). The percentages of motile and progressively motile sperm cells were measured (2 × 10^6^ spermatozoa per mL) on a Hamilton-Thorne CEROS II Analyzer. Values are based on a parametric paired *t*-test, *p < 0.05. “−” = control group, “+” = POPs-exposed group, *n =* 15 males per generation per treatment.

The reproductive organs weights and sperm parameters (motility, SCSA, CM3) were performed to assess any paternal transmission of POPS-associated anomalies. Early life exposure to POPs induced subfertility through two generations of males (F1 and F2). Relative epididymis and prostate weights at PND 90 were smaller in F1 and F2 from the POPs lineage, whereas no differences were observed in F3 (Figure 3A,B). No changes were noted in testis or seminal vesicle weights in any generation (Figure S2A,B). Daily testicular spermatid production and caudal epididymal sperm counts were lower in F1 and F2 males from the POPs lineage relative to the control lineage but were no longer observed in F3 (Figure 3C,D). The percentages of motile and progressively motile sperm were decreased but only in F1 due to the early life exposure to POPs (Figure 3E, Table S4). In addition, the motility parameters lateral head displacement amplitude (ALH) and the beat cross frequency (BCF) were reduced in F1 and F2 POPs lineage (Table S4). No evidence of compromised sperm DNA integrity or protamine content were apparent (Table S5). Thus, paternal POPs exposure induced mostly intergenerational consequences on the reproductive tract and sperm parameters.

### 3.3. Early-Life Exposure to POPs Induced Modifications of the Sperm DNA Methylome Linked to Human Diseases

**Figure 4:**
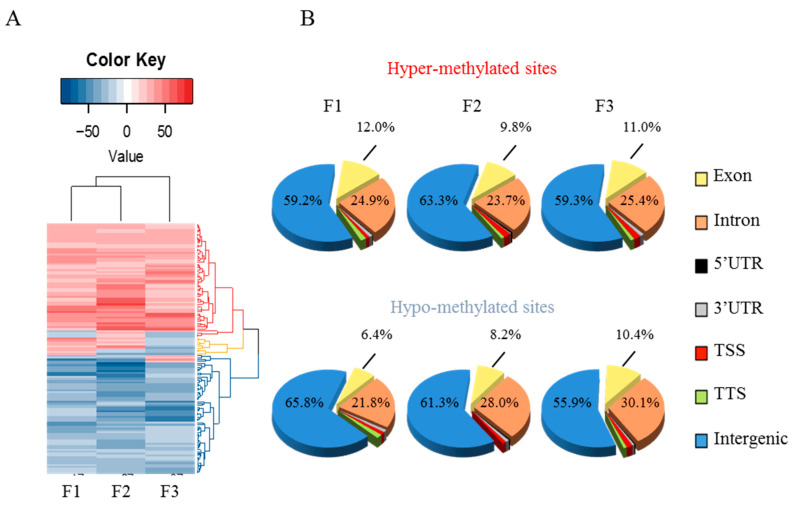
Paternal early life exposure to POPs alters the sperm cell methylome in 2 generations of descendants. Methylation of the sperm cell genome was analyzed in F1, F2 and F3 adults. (**A**) Heat map of methylation level per site based on hierarchical cluster analysis using Euclidean distance measurement of differential methylation expressed as a percentage. Only sites with data over the three generations are included. (**B**) Pie charts of the distribution of differentially methylated sites among 7 categories of genomic location based on overlapping of CpG locus position with annotated genomic features as assessed using Homer v4.8. Refseq.

**Figure 5:**
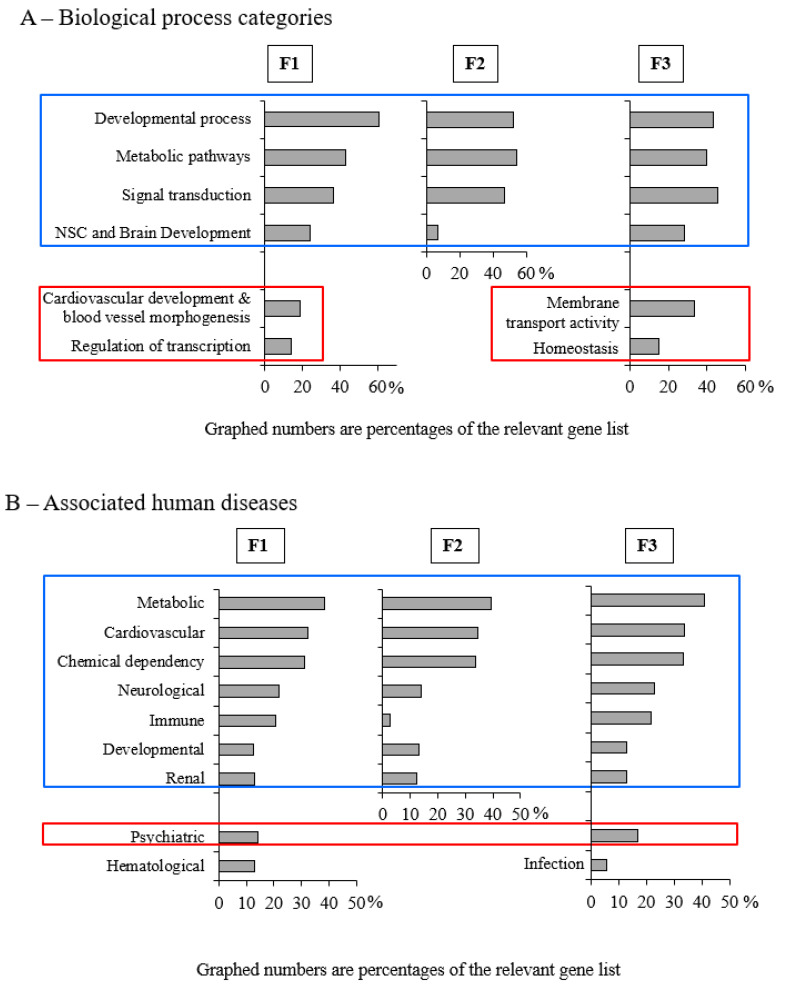
Gene ontology of the differential methylation in F1, F2 or F3 males from POPs lineage. (**A**) Graphic representation of biological process identification (Gene Ontology) excluding intergenic regions (DAVID v6.8, Gene_Ontology: GOTERM_BP_4, Figure 3. *p* < 0.05). (**B**) Associated human disease categories; percentage of genes involved, based on EntrezGene identifiers corresponding to rat differentially methylated sites (excluding intergenic regions, DAVID v6.8. Disease: GAD_disease + GAD_disease_class, *p* < 0.05.).

**Figure 6:**
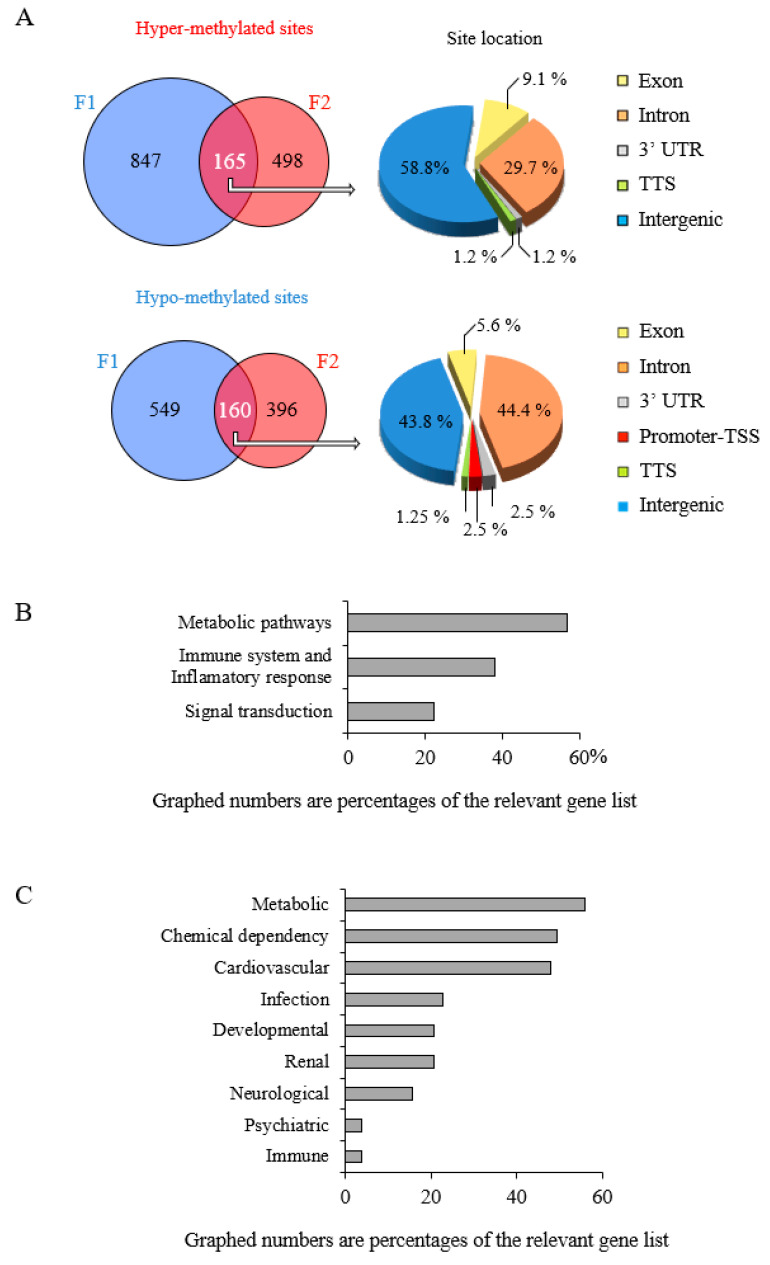
Intergenerational transmission of modifications of the sperm cell methylome of males in POPs lineage. (**A**) Venn diagram of F1 to F2 conservation of differentially methylated sites due potentially to F0 POPs exposure. The locus positioning was analyzed as in Figure 4A. Pie charts show the genomic distributions of the differentially methylated sites. (**B**) Biological processes associated with differentially methylated genes conserved from F1 to F2; (**C**) Human disease associations identified as described in Figure 5. An enrichment *p* value was calculated using DAVID for each pathway term. Pathways were identified as significant at *p* < 0.05 Gene ontology analysis revealed three biological processes that correspond to the affected conserved genes: (1) Metabolic pathways (lipid processes); (2) Immune system (defense) and inflammatory response (complement activation); (3) Signal transduction (response to stress, cell death regulation). Eight categories of human disease are potentially involved: (1) metabolic diseases (hypercholesterolemia, diabetes mellitus type 2); (2) chemical dependency (tobacco use disorder, alcoholism); (3) cardiovascular disease; (4) immune system and infectious disease (natural antibodies, lupus); (5) developmental; (6) renal disease (chronic renal failure, kidney aging); (7) neurological disorders (Alzeimer’s disease); and (8) psychiatric diseases or sleep disorders (Figure 6B,C; Tables S13 and S14).

**Figure 7:**
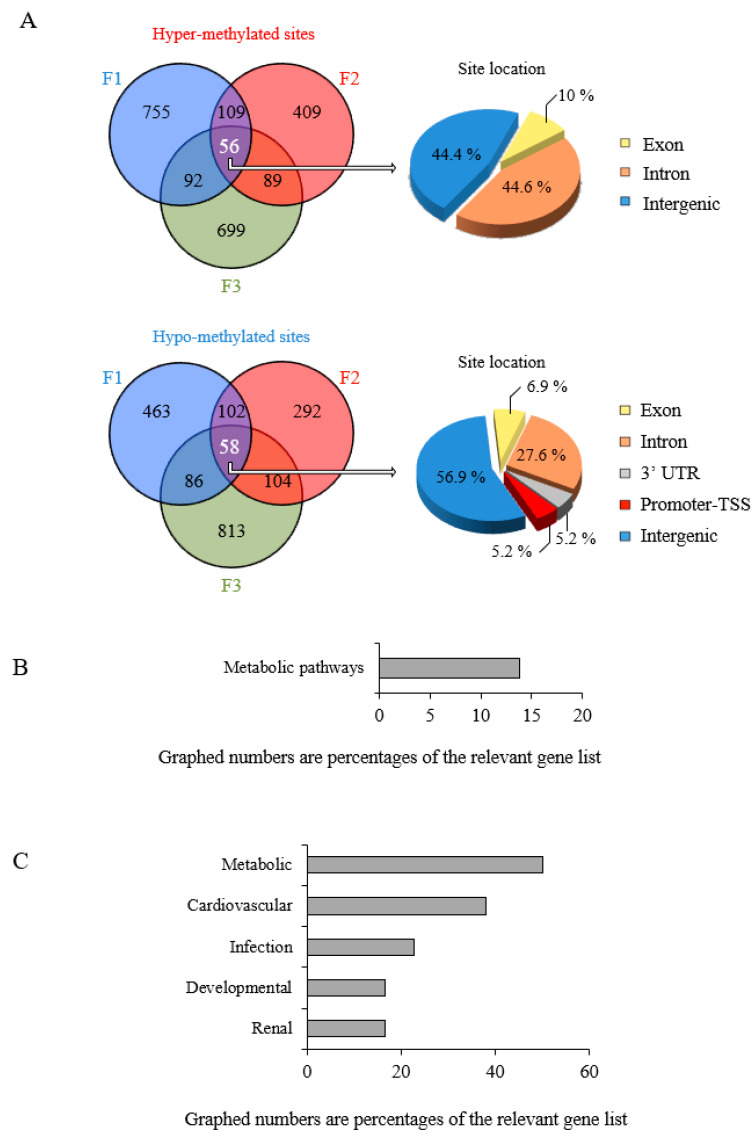
Trans-generational transmission of modifications of the sperm cell methylome of malesin POPs lineage. (**A**) Venn diagram of F1 to F2 to F3 conservation of differentially methylated sites due potentially to F0 POPs exposure. The locus positioning was analysed as in Figure 4A. Pie charts show the genomic distributions of the differentially methylated sites. (**B**) Biological processes associated with differentially methylated genes conserved from F1 through F2 to F3; (**C**) Human disease associations identified as described in Figure 5. An enrichment *p* value was calculated using DAVID for each pathway term. Pathways were identified as significant at *p* < 0.05.

A RRBS assay was performed to detect the sperm DNA methylome modifications then these modifications were confirmed by pyrosequencing. CpG islands are specific methylation enriched regions. This DNA modification occurs primarily at cytosines (C) in cytosine–phosphate–guanine dinucleotides (CpG) allowing the regulation of gene expression. By reduced representation bisulphite sequencing (RRBS) differentially methylated sites (DMS) were detected in each generation in order to analyzed the methylation profiles of rat epididymal sperm across 3 generations (6 cases and 6 controls per generation). Significant DMS were identified as sites having FDR below 1% and only CpG with a minimum coverage of 10 reads and a methylation difference of 20% have been retained. Over-methylation and under-methylation are shown in Figure 4, Figure 5, Figure 6 and Figure 7. Pyro-sequencing validation of DNA methylation pattern on specific genes observed in RRBS shows that the hyper-methylated Tbx2 and hypomethylated Rapsn sites in F1 returned to control levels in F2 and F3 progeny as expected (Figures S3 and S4). The heat-map representation of methylation obtained by hierarchical clustering (Figure 4A) shows slightly greater similarity between F1 and F2 but overall similarity between the three generations. Based on principal component analysis, there was little to discriminate between the two treatments, indicating that POPs had no pronounced effect overall on the rat sperm cell genome (Figure S5). However, this does not exclude strong local effects, as observed using phenotype and bioinformatics analysis. With our collaborator, we participated to the development of a novel permutation method (methylInheritance) which allows the evaluation of the significance level of the number of conserved DMS across several generations. Using permutation analysis, it was confirmed that the pattern conserved across generations was not due to random methylation but is associated with an effect of POPs treatment [35]. Based on the overlap of CpG sites, Refseq gene annotations were segregated into 7 categories: promoter (−2 kb, +100 bp), TTS, exonic, intronic, 5′ UTR, 3′ UTR and intergenic regions. The distribution of the differentially methylated sites (Figure 4B) did not vary widely, with intergenic regions dominating, followed by introns and exons, and less than 4% in UTR, promoter and TTS regions.

Potential cross-generational effects of POPs on biological process and pathologies (after conversion of identifiers from rat to human) were examined using functional analysis of the differentially methylated genes in genic regions (excluding intergenic associated genes) by Gene Ontology [48] using DAVID version 6.8. The modifications conserved over the 3 generations are associated with 4 main biological process categories: (1) developmental processes (brain, kidney, respiratory system, embryo) and cell proliferation; (2) metabolic pathways (primarily lipid and carbohydrate metabolism); (3) cell–cell signal transduction, hormonal response and cell death regulation; and (4) nervous system central (NSC), brain development and learning and synaptic transmission (Figure 5A, Tables S7, S9 and S11). Deregulation of genes involved in the blood system and vascular development (coagulation, angiogenesis) and in transcription regulation (gene expression, RNA metabolism) was apparent in F1. Membrane transport and homeostasis appeared in F3, whereas F2 had no distinctive features. Seven categories of human diseases are associated with these genes: (1) metabolic diseases (diabetes types 1 and 2, hypercholesterolemia); (2) cardiovascular diseases (cardiomyopathy, hypertension); (3) chemical dependencies (tobacco use disorder); (4) neurological diseases (Alzeimer’s, stroke); (5) immune diseases (natural antibodies); (6) developmental diseases (oral cleft, eclampsia, bone density); (7) renal disease or chronic renal failure (Figure 5B, Tables S8, S10 and S12). Some specific gene-disease associations appeared in F1 and F3 but not in F2: bipolar or major depressive psychiatric disorders. Haematological diseases involving erythrocytes or hematocrit were associated only with F1, while lupus and infectious disease (hepatitis C) were associated only with F3. The gene differential methylations observed in this animal study and their associations with human disease appear to corroborate epidemiological studies of relationships between POPs and health problems among the Inuit.

Of particular interest are genes encoding DNA-methyltransferase. Exon 10 of *Dnmt3l* was found hyper-methylated in F1 but not in F2 or F3 (Table S6). Although DNMT3L does not have a catalytic subunit, it is an important co-activator of DNMT3a and B activity and *Dnmt3l* knockout has been shown to impair spermatogenesis and cause male infertility [49,50], a phenotype observed in F1. In fact, at decreased stringency (basic settings of 10 reads per sample, *q* value ≤ 0.01 and ±≥10% average difference, COV10_Met10), *Dnmt3l* is still hyper-methylated in F1 but was found hypo-methylated in F2 and F3. This raises the possibility that direct exposure to POPs can modify the methylation of a key methylation enzyme gene, which might be reversed in subsequent generations.

### 3.4. Epigenetic Marks Modified by POPs Are Heritable

The previous results were used to localize which part of the methylome was modified among generations to determine the heritability of the epigenetic marks. The conservation of differential methylation between F1 and F2 is shown in Venn diagrams (Figure 6A). Non-negligible proportions of the modifications counted in F1 are carried to F2 via paternal transmission. Although hyper-methylated sites are more numerous than hypo-methylated sites in F1, the proportion of hypo-methylated sites conserved between F1 and F2 in intronic regions is greater, whereas the overall distributions of hyper-methylated conserved sites in F1, F2, F3 are similar (Figure 6A and Figure S6A). This greater transmission of intronic hypo-methylation from F1 to F2 has been observed previously in epidemiological studies of the association between exposure to POPs and DNA methylation [51,52,53]. Intronic hypo-methylation implies unmasking of promoter, enhancer or regulatory regions controlling adjacent genes [54,55], resulting in genomic instability [56,57].

The same analysis revealed nearly equal numbers of hyper-methylated and hypo-methylated sites conserved through F1, F2 and F3 (Figure 7A). POPs-induced epigenetic modifications of the sperm cell genome may thus be trans-generational. Unlike between F1 and F2, intronic hyper-methylation was transmitted stably across the 3 generations, while the distribution of the hypo-methylated sites was quite similar to what was observed in F1, F2 or F3 (Figure 7A and Figure S7A). Hyper-methylation of intronic sequences is known to modulate transcriptional activity of target genes [58]. Gene Ontology analysis revealed that only genes relating to metabolism (particularly lipid) retained their epigenetic modifications across the 3 generations. The associated human disease categories are (1) metabolic disorders (hypercholesterolemia, diabetes); (2) cardiovascular disease; (3) infectious disease; (4) developmental skeletal system disorders; (5) chronic renal failure and kidney aging (Figure 7B,C, Tables S15 and S16).

## 4. Discussion

Our study is one of the first report demonstrating that early-life exposure to environmentally relevant contaminants can alter the sperm cell methylome and affect development in more than one generation of descendants. Novel aspects of our study include the use of a mixture of organic compounds that is representative of that to which humans are exposed in the Canadian arctic and isolation of paternal lineage effects using an outbred line of Sprague Dawley rats mated with unexposed females and limited inbreeding. Based on the observed developmental disorders and placental defects, reduced fetal growth, neonatal and postnatal mortality, congenital defects and delayed puberty, the compounds appear to produce effects that are in some cases strikingly similar to those experienced in Inuit [9,11,12]. This experimental approach may represent a good prognostic tool for anticipating human health problems that could arise from exposure to these organic pollutants, particularly in the Inuit population.

Perturbation of regulators that are essential for establishing or maintaining a normal epigenome and correct gene expression is believed to underlie numerous pathologies [21,23,58,59,60].

Our results show that early-life exposure to the POPs mixture induces subfertility through two generations of males (F1 and F2) and has a direct and deleterious effect on sperm quality in F1. This reduced fertility could be due to sperm cell quantity as well (Figure 3C–E, Table S4). Another effect appears to be fewer fertilized oocytes in untreated females and possibly greater preimplantation losses (Figure 1E). Declining human fertility due in part to paternal exposure to xenobiotics has been suggested before [61,62]. Although the fertility of the Inuit population seems stable, this is based on live births per female with no consideration of paternity or the age of the population [9]. No reliable data exist on the fertility of Canadian Inuit men. No reduction in fertility has been shown in Greenland Inuit couples exposed to high levels of POPs [63]. The putative deleterious effects of consuming POP-contaminated marine animals may be outweighed by the positive effects of other constituents of this type of food, such as antioxidants and n-3 polyunsaturated fatty acids, which were not present in the rat diets used in our study. There are thus no data on Inuit male fertility to compare with the effects of POPs on male lineage fertility in our study.

The lower F2 birth weight and persistent lower body weight in the POPs-exposed lineage appeared to be associated with lower placenta mass and hence restriction of fetal growth due to reduced nutrient and oxygen uptake [64,65]. In humans, placental abnormalities are associated with intrauterine growth restriction (IUGR), which leads to serious complications such as low birth weight [66]. In other species such as sheep, abnormal placenta and fetal growth restriction lead to reduced testosterone production and delayed onset of puberty [67], two phenotypes observed in F1 and F2 POPs-lineage rats (Figure 2D,E). However, the mechanisms that determine the onset of puberty are complex and associated with a testosterone surge accompanied by neuro-hormones such as gonadotropin-releasing hormone (GnRH), follicle-stimulating hormone (FSH), luteinizing hormone (LH), insulin-like growth factor 1 (Igf1), leptin, growth hormones and kisspeptins [68,69]. These observations have to be correlated with epidemiological studies in Inuit populations in Canada that show 3 times more infant mortality with 10% due to an abnormality of the placenta, 9% due to growth delay and another 9% due to developmental abnormalities than non-aboriginal Canadian [9,11]. Additionally, a study of pregnant Inuit women from Arctic Quebec revealed that prenatal exposure to POPs is correlated to a shorter pregnancy duration, which, in turn, is associated with reduced infant birth weight, length and head circumference [12].

In Inuit communities in Greenland, Alu and LINE-1 assays of blood leukocyte DNA have revealed differences in overall DNA methylation in association with exposure to increased concentrations of POPs [52]. These modifications might reflect modifications of the male germline epigenome. In humans and animals, POPs appear to play a role in perturbing the fetal development, therefore it was not surprising to find altered methylation in a class of genes involved in developmental diseases, sudden infant death and eclampsia (in F2 and F3), but also intergenerational and trans-generational conservation of methylation patterns in some genes (skeletal system development). A common feature shared by all generations was methylation of genes involved in the immune system and infectious diseases (asthma, lupus, psoriatic, Figure 5B, Figure 6C and Figure 7C; Tables S8, S10, S12, S14 and S16). As shown in a mouse study of trichloroethylene in drinking water [70], our rat model showed differentially methylated genes associated with lupus disease. It is well established that some POPs can affect respiratory system development, leading to allergies, hypersensitivity and an impaired immune system. Canadian Inuit children present frequent respiratory tract infections leading potentially to respiratory morbidity later in life [14,71]. Based on differential methylation of genes, the effects of the POPs mixture on metabolic pathways and in particular predisposition to lipid and cholesterol-associated diseases and types 1 and 2 diabetes appeared transmissible via the paternal lineage across three generations of rats, as seen for long-term complications of diabetes such as cardiovascular disease (coronary, cardiomyopathy, hypertension, arteriosclerosis) and renal disease (chronic and kidney failure). POPs have been implicated previously in the development of obesity, dyslipidemia and insulin resistance, all of which are precursors of type-2 diabetes and cardiovascular disease [72,73,74]. The incidence of diabetes and obesity associated with cardiovascular disease has been increasing for years in the Inuit population [75]. More recently, PCBs and p,p’-DDE have been associated with increased risk of diabetes [76]. The genes identified in our study suggest a novel line of research on diabetes and its cardiovascular and renal complications.

Persistent organic pollutants are known to induce neurological and nervous system disorders [9,11]. This appears among Inuit children in the form of scholastic difficulties, poor short-term and visual recognition memories and delays in cognitive development. In addition to genes associated with Alzheimer’s disease, which decreases the ability to focus on, recall or organize information, we found methylation of other genes that are usually expressed in the brain (Table S17), such as *Bai2* involved in spatial and learning memory [77], *Dbx1* involved in innate behaviour [78], *Gbx1* and *Gbx2* in memory and learning [79,80] or *Stxa1* in working memory and attention deficit/hyperactive disorder [81]. Methylation of these genes could be markers of neurological pathologies associated with exposure to POPs. Secondary effects associated with these genes include depressive phenotypes, anxiety or apathy, as observed in Alzheimer’s disease. Some of the methylated genes are associated with psychiatric disorders such as depressive, sleep or bipolar disorder. Based on the reported association of POPS with long-term depression [82,83], the possibility of a role in the high incidence of anxiety and suicidal depression in Inuit communities needs to be investigated. 

In summary, our animal model provides compelling evidence that early-life exposure to organic pollutants found in the Canadian arctic can alter the sperm cell methylome and cause gestational perturbations and developmental defects in more than one generation of male descendants. The implications of this sort of differential methylation in humans need to be studied. From an ecological point of view, the sperm cell epigenomes of wildlife in contaminated and non-contaminated regions need to be compared. Paternal transmission of epigenomes modified by such pollution could play a central role in the evolution of species. 

## Data Availability

All RRBS dataset are available through Expression Omnibus accession numbers GSE109056.

## Supplementary Materials

The following are available online at https://www.mdpi.com/article/10.3390/epigenomes5020010/s1. Figure S1: Body mass of male descendants of females exposed to POPs, relative to the control group; Figure S2: Exposure of females to POPs, effects on fertility parameters in the male descendants; Figure S3: The percentage of hypo-methylated sites in intronic regions is greater in intergenerational transmission; Figure S4: The percentage of hyper-methylated sites in intronic regions is greater in trans-generational transmission; Table S1: Composition of the mixture of persistent organic pollutant (POP) compounds used in this study; Table S2: Growth and fertility parameters of the F0 founder females; Table S3: Development of ovarian follicles in F0 founder females and their F1 offspring; Table S4: Sub-fertility of sperm of descendants of F0 founders exposed to POPs; Table S5: Sperm chromatin stability assay and DNA/protein composition; Table S6: Differentially methylated Dnmt genes in F1, F2 and F3 generations of male Sprague Dawley rats after F0 maternal exposure to persistent organic pollutants; Tables S7, S9 and S11: Biological processes associated with genes found differentially methylated in sperm cells of F1, F2 and F3 progeny of female rats exposed to persistent organic pollutants; Tables S8, S10 and S12: Human diseases corresponding to genes found differentially methylated in sperm cells of F1, F2, and F3 offspring of females rats exposed to persistent organic pollutants; Table S13: Differentially methylated genes conserved in sperm cells of both F1 and F2 progeny of female rats exposed to persistent organic pollutants; Table S14: Human diseases corresponding to genes found methylated differentially in sperm cells of both F1 and F2 progeny of female rats exposed to persistent organic pollutants; Table S15: Biological processes associated with genes found methylated differentially in sperm cells of F1, F2 and F3 progeny of female rats exposed to persistent organic pollutants; Table S16: Human diseases corresponding to genes found differentially methylated in sperm cells of F1, F2 and F3 progeny of female rats exposed to persistent organic pollutants; Table S17: Genes with brain-specific expression found differentially methylated in sperm cells of descendants of female Sprague Dawley rats exposed to POPs. Table S18: The quality of sequenced reads for each sample (on library per sample); Table S19: The quality of alignment for each sample (one library per sample); Table S20: Statistics related to the coverage of the CpG sites identified in each sample.
