# Supplementary material for: Early-Life Exposure to Environmental Contaminants Perturbs the Sperm Epigenome and Induces Negative Pregnancy Outcomes for Three Generations via the Paternal Lineage
Source: Epigenomes. 2021 May 1;5(2):10. doi: 10.3390/epigenomes5020010 (PMC8594730; doi:10.3390/epigenomes5020010)
Supplement: Supplementary file 1 [file epigenomes-05-00010-s001.zip › epigenomes-1172457-supplementary.pdf]

Maurice *et al.* Supplementary data, Figure S1

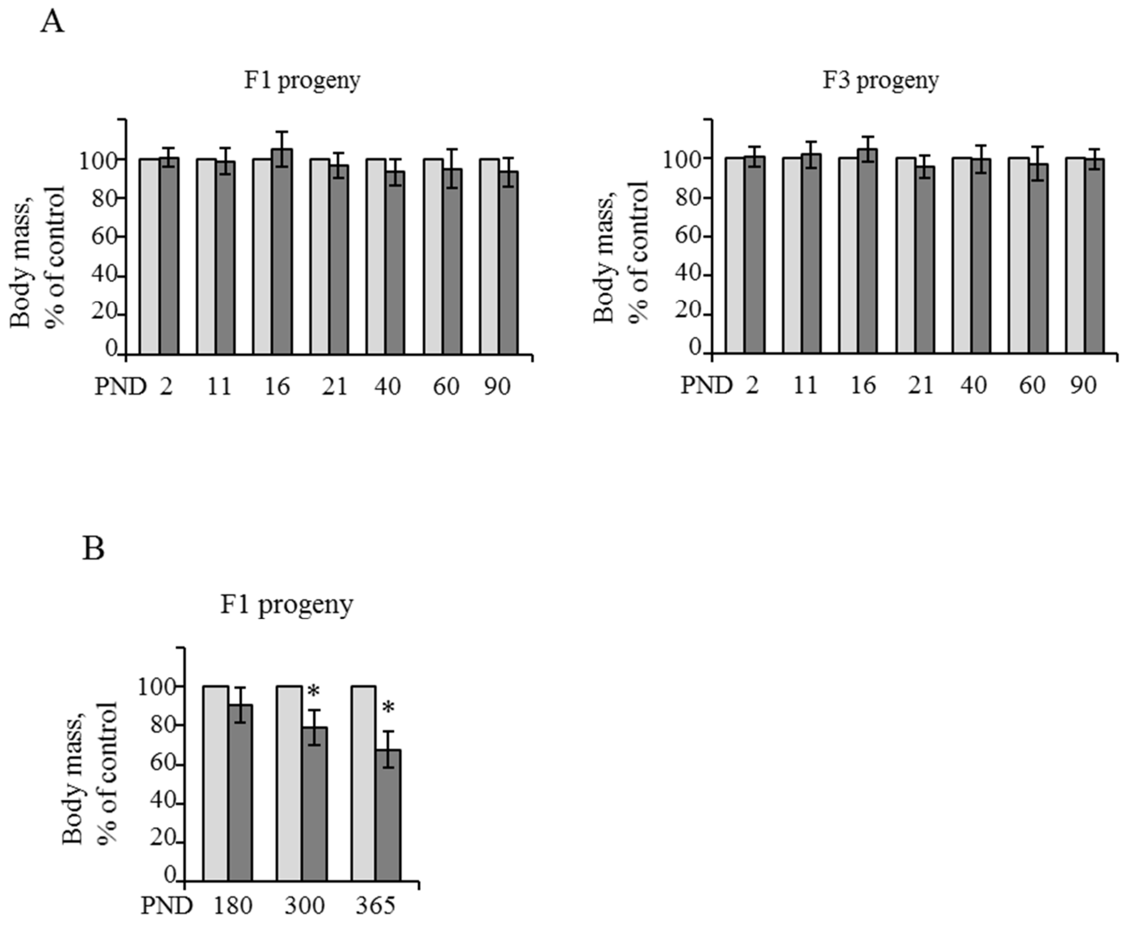

Figure S1. Body mass of male descendants of females exposed to POPs, relative to the control group.

(A) F1 and F3 progeny; (B) F3 progeny for up to 1 year; light grey = control group; dark grey = exposed group.

Maurice *et al.* Supplementary data, Figure S2

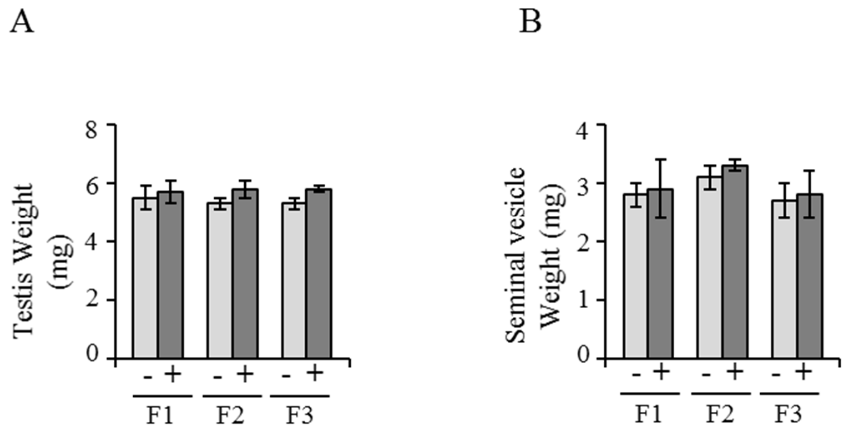

Figure S2. Exposure of females to POPs, effects on fertility parameters in the male descendants

A) Body-mass corrected testis wet mass; B) Body-mass adjusted seminal vesicle mass; Values are means with s.e.m. from a parametric paired t-test; "-" is unexposed, "+" is exposed; n = 15 animals per treatment per generation (*P < 0.05)

Maurice *et al.* Supplementary data, Figure S3

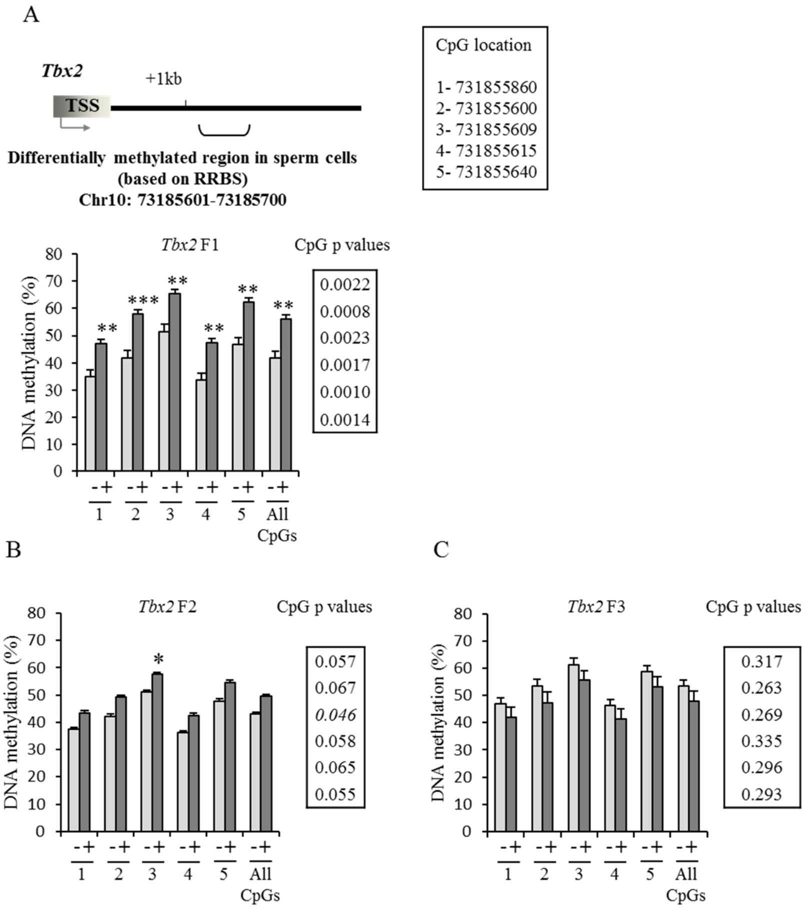

Figure S3. Pyro-sequencing validation of RRBS data in the Tbx2 gene

Pyrosequencing after bisulphite conversion shows the methylation levels of 5 CpG regions located in the Tbx2 gene in F1 (A), F2 (B) and F3 (C) descendants of Sprague Dawley F0 female rats exposed (+) or or not (-) to POPs. Standard error of the mean was calculated within generations (unexposed vs. exposed group) using a parametric paired t-test. *P < 0.05, **p < 0.01, ***P < 0.001.

Maurice *et al.* Supplementary data, Figure S4

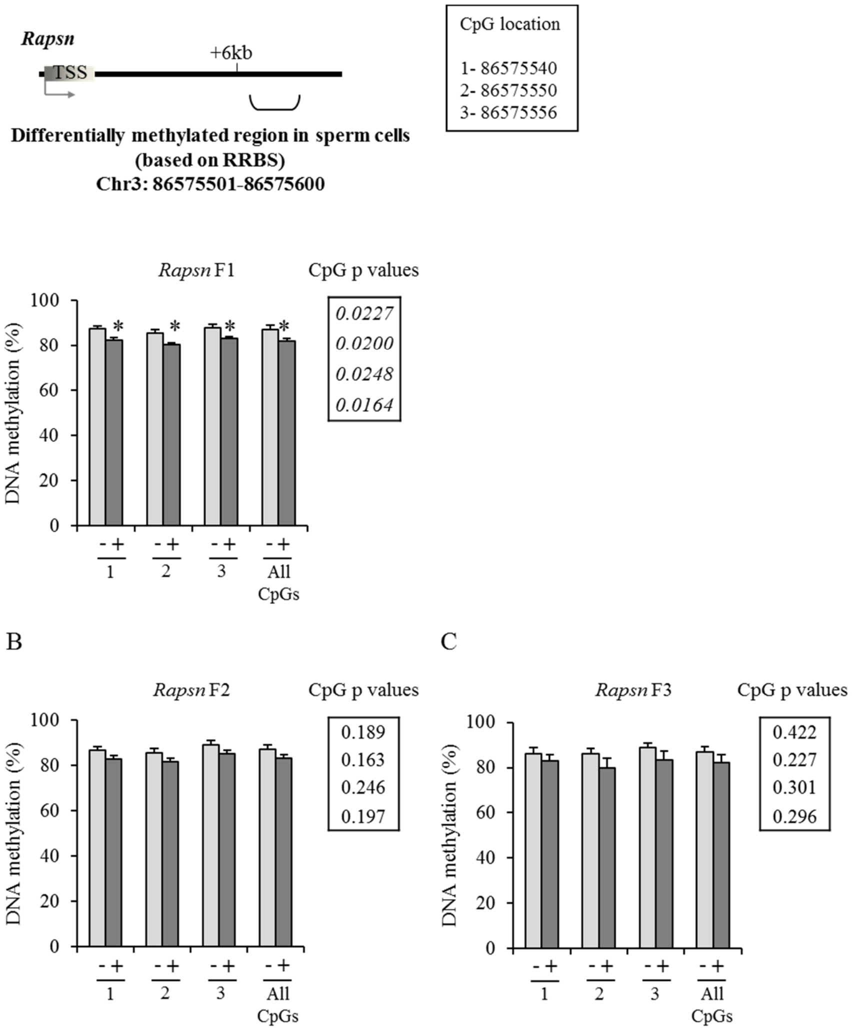

Figure S4. Pyro-sequencing validation of RRBS data in the Rapsn gene

Pyrosequencing after bisulphite conversion shows the methylation levels of 5 CpG regions located in the Tbx2 gene in F1 (A), F2 (B) and F3 (C) descendants of Sprague Dawley F0 female rats exposed (+) or or not (-) to POPs. Standard error of the mean was calculated within generations (unexposed vs. exposed group) using a parametric paired t-test. *P < 0.05, **P < 0.01, ***P < 0.001.

Maurice *et al.* Supplementary data, Figure S5

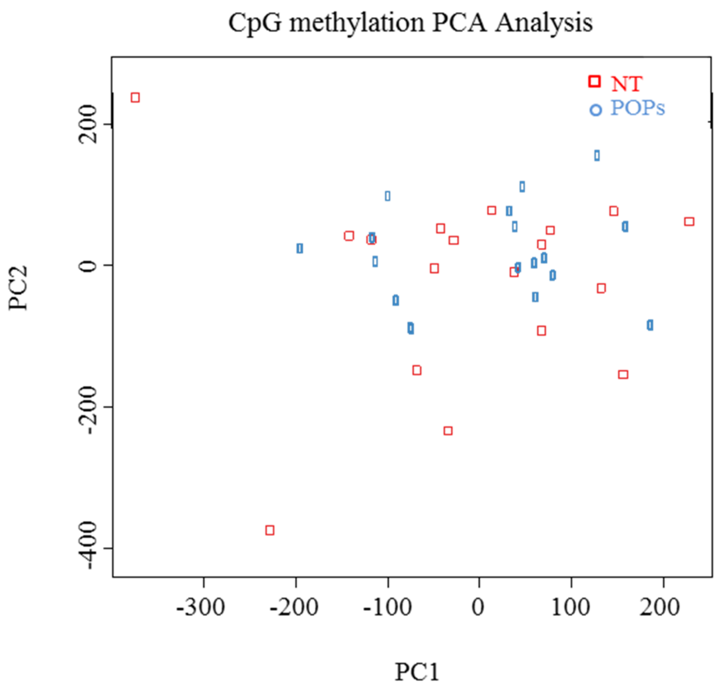

Figure S5. Principal component analysis of methylation values

Principal component analysis of CpG site methylation values for unexposed (square, n = 18) and exposed (circle, n = 18) Sprague Dawley rat male to POPs via F0 females. All data were normalized and PCA was performed using methylKit version 0.9.4. Shown are the first and second principal components accounting for the most variance in the normalized dataset.

Maurice *et al.* Supplementary data, Figure S6

A

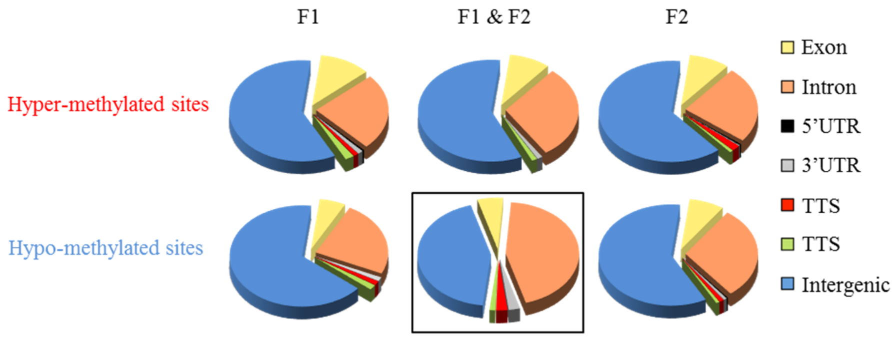

B

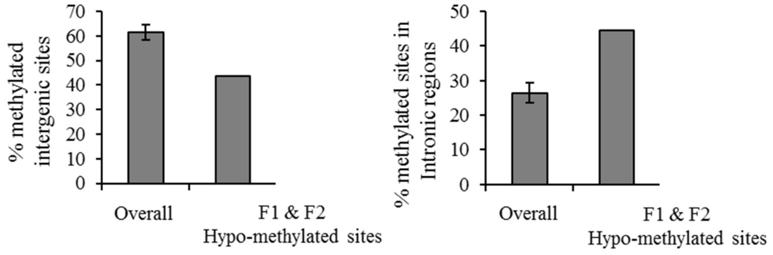

Figure S6. The percentage of hypo-methylated sites in intronic regions is greater in intergenerational transmission.

A) Venn diagrams of hyper-methylated site (top) and hypo-methylated site (bottom) distribution throughout the genome in F1, F2 or both generations. B) Methylated sites in intergenic regions (left) or intronic regions (right). "Overall" represents the average from all Venn diagrams without F1 and F2 hypo-methylated sites.

Maurice *et al.* Supplementary data, Figure S7

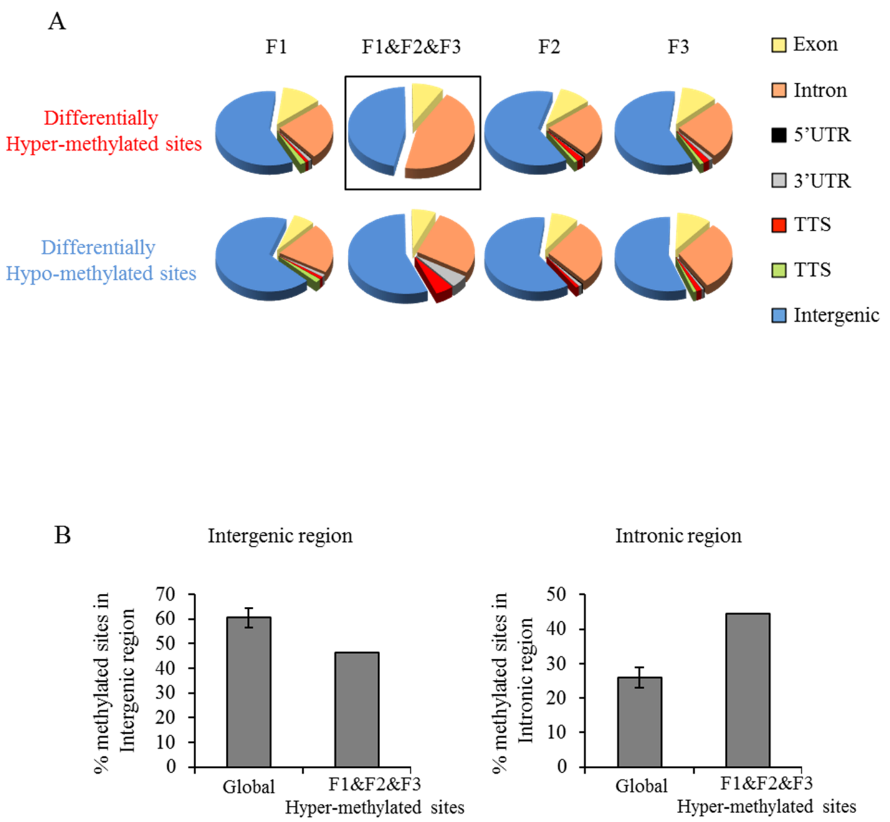

Figure S7. The percentage of hyper-methylated sites in intronic regions is greater in trans-generational transmission.

A) Venn diagrams of hyper-methylated site (top) and hypo-methylated site (bottom) distribution throughout the genome in F1, F2, F3 or all three generations. B) Methylated sites in intergenic regions (left) or intronic regions (right). "Overall" represents the average from all Venn diagrams without F1, F2 and F3 hypo-methylated sites.

Maurice *et al.* Supplementary data, Table S1

Table S1. Composition of the mixture of persistent organic pollutant (POP) compounds used in this study.

Compound	Source[b]	% Weight	Dose administered (μg/kg body weight/3 times per week)	
			Control group	POPs group
Aroclor and congener neat mix[a]	AccuStandard	32.4	0	500.00
Technical chlordane	AccuStandard	21.4	0	330.3
p,p′-Dichlorodiphenyldichloroethylene	Sigma-Aldrich	19.3	0	297.8
p,p′-Dichlorodiphenyltrichloroethane	SigmaAldrich	6.8	0	104.9
Technical toxaphene	AccuStandard	6.5	0	100.0
a-Hexachlorocyclohexane	Sigma-Aldrich	6.2	0	95.7
Aldrin	Sigma-Aldrich	2.5	0	38.6
Dieldrin	Sigma-Aldrich	2.1	0	32.4
1, 2, 4, 5-Tetrachlorobenzene	Sigma-Aldrich	0.9	0	13.9
p, p′-Dichlorodiphenyldichloroethane	Sigma-Aldrich	0.5	0	7.7
β-Hexachlorocyclohexane	Sigma-Aldrich	0.4	0	6.2
Hexachlorobenzene	AccuStandard	0.4	0	6.2
Mirex	Sigma-Aldrich	0.2	0	3.1
Lindane	Sigma-Aldrich	0.2	0	3.1
Pentachlorobenzene	Sigma-Aldrich	0.2	0	3.1

aMix containing Aroclor 1260 (58.9%), Aroclor 1254 (39.3%), 2,4,4'-trichlorobiphenyl (PCB 28; 1.0%), 2,2',4,4'-tetrachlorobiphenyl (PCB 47; 0.8%), 3,3',4,4',5-pentachlorobiphenyl (PCB 126; 0.02%), and 3,3',4,4'-tetrachlorobiphenyl (PCB 77; 0.004%). CAS number of each substance are presented in previous study [39].
bSigma-Aldrich (Oakville, ON); AccuStandard (New Haven, CT).

Maurice *et al.* Supplementary data, Table S2

Table S2. Growth and fertility parameters of the F0 founder females (n=4).

Parameter	Control	POPs
F0 body weight after 5 weeks of gavage (g)	282 ± 11	303 ± 15
F0 body weight at parturition (g)	464 ± 20	482 ± 12
F0 body weight at weaning (g)	323 ± 11	339 ± 20
Fertility rate (%)[a]	100	100
Gestation index (%)[b]	100	100
Gestational length (days)	22 ± 0	22 ± 0
Males (%)	47 ± 8	55 ± 7

aFertility rate was the number of gravid females / the number of mated females x 100.
bGestation index was the number of females with live births / the number of gravid females x 100.

Table S3. Development of ovarian follicles in F0 founder females and their F1 offspring.

Follicule count	F0 founders (n=4)		F1 offspring (n=4)	
	Control	POPs	Control	POPs
Primordial	72 ± 5	77 ± 3	71 ± 2	71 ± 2
Primary	17 ± 4	15 ± 3	18 ± 2	16 ± 1
Preantral	5 ± 1	5 ± 1	6 ± 1	7 ± 1
Antral	6 ± 2	4 ± 0	5 ± 1	5 ± 1

Maurice *et al.* Supplementary data, Table S4

Table S4. Sub-fertility of sperm of descendants of F0 founders exposed to POPs (n=15).

Generation	F1		F2		F3	
Treatment	Control	POPs	Control	POPs	Control	POPs
**Flow cytometer**						
% live sperm cells	56 ± 1	57 ± 1	63 ± 1	57 ± 1	71 ± 1	73 ± 3
% sperm cells with intact acrosomes	94 ± 1	93 ± 1	94 ± 1	93 ± 1	91 ± 2	94 ± 2
% live sperm cells with intact acrosomes	57 ± 1	57 ± 1	57 ± 1	57 ± 1	70 ± 2	71 ± 3
**CTC pattern**						
% F pattern	74 ± 1	75 ± 1	81 ± 1	83 ± 1	88 ± 2	85 ± 3
% B pattern	21 ± 1	21 ± 1	19 ± 1	17 ± 1	12 ± 1	15 ± 1
% AR pattern	3 ± 1	0	0	0	0	0
**Motility parameters**						
% motile	60 ± 4	**33 ± 4***	62 ± 3	63 ± 4	72 ± 4	77 ± 5
% displaying progressive motility	53 ± 3	**29 ± 5***	58 ± 3	60 ± 4	68 ± 3	74 ± 3
VAP (µm/sec)	185.8 ± 4.5	179.1 ± 4.7	184.8 ± 4.5	183.1 ± 4.7	183.3 ± 4.4	186.6 ± 2.1
VSL (µm/sec)	116.4 ± 3.1	112.1 ± 3.3	115.4 ± 3.1	114.1 ± 3.3	113.3 ± 3.2	116.3 ± 2.5
VCL (µm/sec)	322.5 ± 6.9	314.7 ± 7.1	321.5 ± 6.9	318.7 ± 7.1	318.3 ± 4.3	316 ± 2.8
ALH (µm)	17.7 ± 0.2	**16.5 ± 0.3***	17.4 ± 0.2	**16.1 ± 0.3***	16.4 ± 0.4	16.9 ± 0.5
BCF (Hz)	20.1 ± 0.3	**18.3 ± 0.3***	21.1 ± 0.3	**19.3 ± 0.3***	20.4 ± 0.5	20.1 ± 0.1
% STR	61.3 ± 0.5	60.3 ± 0.8	63.1 ± 0.5	62.1 ± 0.8	62.4 ± 0.6	63.1 ± 0.4
% LIN	36.4 ± 1.0	36.9 ± 1.0	35.9 ± 1.0	36.1 ± 1.0	35.9 ± 0.7	36.3 ± 0.4

*statistically significant from control group $P<0.05$

Table S5. Sperm chromatin stability assay and DNA/protein composition

	% DFI		% CMA3 positive		% CMA3 negative	
**Generation**	**Control**	**Exposed**	**Control**	**Exposed**	**Control**	**Exposed**
**F1**	1 ± 0	1 ± 0	0	0	100	100
**F2**	1 ± 0	1 ± 0	0	0	100	100
**F3**	1 ± 0	1 ± 0	0	0	100	100

The Sperm Chromatin Structure Assay (SCSA®) was used to measure sperm chromatin fragmentation. The chromomycin A3 (CMA3) assay was used to quantify protamine (positive = low, negative = abundant). n = 4 in F1, F2 and F3 control and exposed groups. DFI = DNA fragmentation index

Maurice *et al.* Supplementary data, Table S6

Table S6. Differentially methylated *Dnmt* genes in F1, F2 and F3 generations of male Sprague Dawley rats after F0 maternal exposure to persistent organic pollutants

COV10_Met20

Symbol	Name	Generation	Methylation status	Location
*Dnmt*3l	DNA-methyltransferase 3-like	F1	Hyper	Exon 10

COV10_Met10

Symbol	Name	Generation	Methylation status	Location
*Dnmt*3l	DNA-methyltransferase 3-like	F1	Hyper	Exon 10
		F2	Hypo	Intron 6
		F3	Hypo	Exon 7

Upper panel: methylKit version 0.9.4 parameters = 10 reads per sample, q value <= 0.01 and ± ≥ 20% average differences between groups of replicates; lower panel: methylKit version 0.9.4 parameters = 10 reads per sample, q value <= 0.01 and ± ≥ 10% average differences between groups of replicates.

Maurice *et al.* Supplementary data, Table S7

Table S7. Biological processes associated with genes found differentially methylated in sperm cells of F1 progeny of female rats exposed to persistent organic pollutants.

Process or function	Count (total = 397)	%	P value
**Developmental**	242	60.95	
**cellular processes**			
cell development	86	18.7	5.77E-05
cell morphogenesis	70	15.2	2.89E-07
cell motility	46	10.0	1.50E-02
**organ development**	97	21.0	1.28E-02
brain	26	5.6	4.41E-02
tube	25	5.4	2.04E-02
kidney	16	3.5	3.59E-03
renal system	16	3.5	5.85E-03
respiratory system	12	2.6	2.34E-02
lung	11	2.4	2.17E-02
**system development**	138	29.9	1.14E-03
**embryo development**	37	8.0	1.55E-02
**Metabolic**	170	42.82	
positive regulation of metabolism	112	24.3	1.52E-02
negative regulation of metabolism	82	17.8	3.95E-02
organophosphate pathways	44	9.5	1.24E-03
ribose phosphate pathways	22	4.8	1.46E-02
phospholipid metabolism	17	3.7	2.38E-02
inositol phosphate metabolism	7	1.5	6.72E-03
**Signal transduction**	145	36.52	
regulation of cell communication	94	20.4	2.60E-02
intracellular signal transduction	90	19.5	2.76E-03
cell-cell signaling	54	11.7	4.78E-05
response to growth factor	43	9.3	1.82E-04
response to hormone	41	8.9	4.81E-03
signal transduction by protein phosphorylation	36	7.8	2.74E-03
MAPK cascade	35	7.6	2.48E-03
**NSC and brain development**	96	24.18	
nervous system development	88	19.1	1.11E-04
neuron development	62	13.4	2.06E-07
central nervous system development	33	7.2	3.76E-02
learning	14	3.0	5.32E-05
synapse assembly	9	2.0	1.86E-02
memory	8	1.7	1.35E-02
**Regulation of transcription**	56	14.10	
positive regulation of gene expression	56	12.1	4.35E-02
positive regulation of RNA metabolic process	51	11.1	1.92E-02
**Blood system and vascular development**	74	18.63	
circulatory system development	37	8.0	9.60E-03
cardiovascular system development	37	8.0	9.60E-03
blood coagulation	30	6.5	1.35E-04
blood vessel morphogenesis	20	4.3	3.78E-02
regulation of blood circulation	18	3.9	1.43E-03
regulation of angiogenesis	12	2.6	1.75E-02

Based on Gene Ontology (DAVID v6.8, Gene_Ontology: GOTERM_BP_4, functional annotation chart, Count: 2, $p < 0.05$)

Table S8. Human diseases corresponding to genes found differentially methylated in sperm cells of F1 offspring of females rats exposed to persistent organic pollutants.

Disease category	Count (Total = 461)	%	P value
**METABOLIC**	**176**	**38.2**	**1.05E-04**
Cholesterol, HDL	22	4.8	6.57E-03
Triglycerides	20	4.3	2.60E-02
Plasma HDL cholesterol (HDL-C) levels	13	2.8	3.58E-02
Diabetes mellitus type 2	11	2.4	2.15E-02
Type 1 diabetes	5	1.1	4.15E-02
Blood pressure	20	4.3	4.21E-02
Body weight	18	3.9	4.95E-02
Weight gain	9	2.0	2.64E-02
Dyslipidemias	3	0.7	4.58E-02
**CARDIOVASCULAR**	**150**	**32.5**	**4.65E-05**
QT interval	4	0.9	4.91E-02
EK, abnormal	3	0.7	3.35E-02
Coronary disease, myocardial infarction, stroke	3	0.7	2.27E-02
Tunica media	11	2.4	1.55E-02
**CHEMICAL DEPENDENCY**	**143**	**31.0**	**1.18E-07**
Tobacco use disorder	127	27.5	2.72E-08
**NEUROLOGICAL**	**100**	**21.7**	**3.45E-03**
Alzheimer's disease	45	9.8	2.41E-04
Hirschsprung's disease	4	0.9	9.76E-03
**IMMUNE**	**95**	**20.6**	**4.08E-02**
natural antibodies	3	0.7	2.79E-02
**PSYCHIATRIC**	**66**	**14.3**	**4.09E-02**
Sleep disturbances	6	1.3	2.19E-02
Perphenazine	3	0.7	2.27E-02
major depressive disorder	3	0.7	3.35E-02
**RENAL**	**59**	**12.8**	**6.13E-04**
**HEMATOLOGICAL**	**59**	**12.8**	**3.62E-03**
Erythrocyte count	15	3.3	6.58E-04
Hematocrit	8	1.7	3.19E-02
Hemoglobin A, glycosylated	10	2.2	4.71E-02
**DEVELOPMENTAL**	**58**	**12.6**	**3.87E-03**
Cleft lip, cleft palate, tooth abnormalities	7	1.5	2.12E-02
oral clefts	3	0.7	4.58E-02

Based on Gene Ontology using EntrezGene identifiers (DAVID v6.8. Disease: GAD_disease + GAD_disease_class, $p < 0.05$)

Table S9. Biological processes associated with genes found differentially methylated in sperm cells of F2 progeny of female rats exposed to persistent organic pollutants

Process or function	Count (total = 246)	%	P value
**Developmental**	129	52.43	
**cellular process**			
cell differentiation	78	26.6	5.39E-03
cell development	46	15.7	4.33E-02
**Skeletal development**			
skeletal system development	16	5.5	1.03E-02
cartilage development	9	3.1	7.85E-03
**Muscle development**			
muscle organ development	12	4.1	2.47E-02
muscle tissue morphogenesis	7	2.4	4.76E-04
myofibril assembly	4	1.4	4.77E-02
**Connective tissue development**	10	3.4	1.17E-02
**Metabolic**	133	54.06	
positive regulation of metabolism	73	24.9	1.56E-02
regulation of protein metabolism	52	17.7	3.35E-02
regulation of phosphorus metabolism	37	12.6	3.75E-02
organophosphate metabolism	27	9.2	1.38E-02
nucleoside metabolism	12	4.1	2.91E-02
**Enzymatic activity**			
regulation of catalytic activity	47	16.0	4.42E-02
regulation of hydrolase activity	29	9.9	3.17E-02
**Lipid process**			
cellular lipid metabolism	24	8.2	3.29E-02
phospholipid metabolism	12	4.1	2.91E-02
lipid catabolism	10	3.4	3.21E-02
**Carbohydrate metabolism**			
glycosyl compound pathways	13	4.4	1.76E-02
single-organism carbohydrate catabolism	6	2.0	4.74E-02
glucose 6-phosphate pathways	3	1.0	4.43E-02
**Signal transduction**	115	46.74	
intracellular signal transduction	59	20.1	4.47E-03
cell surface receptor signaling pathway	54	18.4	4.11E-02
programmed cell death	39	13.3	4.74E-02
regulation of response to stress	34	11.6	2.47E-02
regulation of cell death	34	11.6	3.52E-02
positive regulation of multicellular organismal process	31	10.6	4.95E-02
response to growth factor	23	7.8	3.42E-02
stress-activated protein kinase signaling cascade	11	3.8	1.38E-02
regulation of endocytosis	10	3.4	3.94E-03
positive regulation of endocytosis	6	2.0	3.32E-02
positive regulation of receptor-mediated endocytosis	4	1.4	2.98E-02
**NSC and brain development**	17	6.91	
positive regulation of nervous system development	13	4.4	3.72E-02
mesenchyme morphogenesis	4	1.4	2.17E-02

Based on Gene Ontology using EntrezGene identifiers (DAVID v6.8. Disease: GAD_disease + GAD_disease_class, $p < 0.05$)

Maurice *et al.* Supplementary data, Table S10

Table S10. Human diseases corresponding to genes found differentially methylated in sperm cells of F2 offspring of females rats exposed to persistent organic pollutants.

Disease category	Count (total = 300)	%	P value
**METABOLIC**	**115**	**39.2**	**1.89E-03**
Triglycerides	18	6.1	1.78E-03
Body height	18	6.1	2.25E-02
Body mass index	16	5.5	2.13E-02
Obesity	14	4.8	1.32E-02
Cholesterol, HDL	14	4.8	4.13E-02
Diabetes mellitus type 2	9	3.1	1.31E-02
Diabetes mellitus type 1	6	2.0	4.11E-02
**CARDIOVASCULAR**	**101**	**34.5**	**2.60E-04**
hypertension	20	6.8	4.07E-02
cardiomyopathy	4	1.4	3.68E-02
Cardiomyopathy, hypertrophic	3	1.0	4.29E-02
Cardiomyopathy, dilated	3	1.0	4.29E-02
**CHEMICAL DEPENDENCY**	**99**	**33.8**	**6.23E-07**
Tobacco use disorder	89	30.4	1.28E-07
**DEVELOPMENTAL**	**38**	**13.0**	**1.97E-02**
**RENAL**	**36**	**12.3**	**2.23E-02**
**AGING**	**26**	**8.9**	**2.58E-02**
kidney aging	6	2.0	2.98E-02
**Neurological**			
Alzheimer's disease	26	8.9	2.64E-02
Apoplexy, cerebral hemorrhages, stroke	7	2.4	3.65E-02
**Immune**			
natural antibodies	3	1.0	1.25E-02
Acquired immunodeficiency syndrome	3	1.0	3.49E-02

Based on Gene Ontology using EntrezGene identifiers (DAVID v6.8. Disease: GAD_disease + GAD_disease_class, $p < 0.05$)

Table S11. Biological processes associated with genes found differentially methylated in sperm cells of F3 progeny of female rats exposed to persistent organic pollutants.

Process or function	Count (total = 411)	%	p value
**Developmental**	117	43.06	
**cellular process**			
cell differentiation	128	26.7	8.37E-04
cell development	77	16.1	9.32E-03
cell morphogenesis	54	11.3	9.98E-03
cell motility	47	9.8	1.71E-02
cell morphogenesis involved in differentiation	41	8.6	1.04E-02
cell projection morphogenesis	40	8.4	2.35E-02
**system development**	136	28.4	8.89E-03
**organ morphogenesis**	35	7.3	3.43E-02
**regulation of cellular component movement**	29	6.1	4.42E-02
**connective tissue development**	12	2.5	4.49E-02
**Metabolic**	163	39.65	
phosphate compound metabolism	104	21.7	1.35E-03
organonitrogen compound biosynthesis	51	10.6	4.65E-03
organophosphate metabolism	42	8.8	6.90E-03
negative regulation of phosphorus metabolism	23	4.8	2.64E-02
glutathione derivative metabolism	5	1.0	8.72E-03
xenobiotic catabolism	3	0.6	2.58E-02
**Lipid metabolism**			
phospholipid	18	3.8	1.59E-02
regulation of	16	3.3	7.69E-03
sphingolipid	9	1.9	3.25E-02
**Enzymatic activity**			
regulation of catalytic activity	74	15.4	4.29E-02
positive regulation of hydrolase activity	35	7.3	6.04E-03
**Carbohydrate metabolism**			
carbohydrate derivative biosynthesis	35	7.3	1.17E-02
**Signal transduction**	188	45.74	
**regulation of cell communication**	100	20.9	1.05E-02
cell surface receptor signaling pathway	93	19.4	3.49E-03
intracellular signal transduction	85	17.7	3.45E-02
cell-cell signaling	58	12.1	8.11E-06
regulation of cell death	52	10.9	4.07E-02
**Hormonal regulation**			
regulation of hormone secretion	15	3.1	1.21E-02
regulation of peptide hormone secretion	14	2.9	5.54E-03
negative regulation of peptide hormone secretion	5	1.0	3.21E-02
response to growth factor	36	7.5	1.92E-02
signal release	21	4.4	7.13E-03
positive regulation of growth	12	2.5	4.60E-02
regulation of fibroblast proliferation	7	1.5	2.32E-02
negative regulation of peptide secretion	5	1.0	3.67E-02
**CNS and brain development**	116	28.22	
nervous system development	80	16.7	9.79E-03
neuron development	44	9.2	2.85E-02
neuron projection development	42	8.8	1.17E-02
synaptic transmission	33	6.9	1.25E-03
neuron projection guidance	30	6.3	4.89E-04
tube morphogenesis	17	3.5	3.03E-02
neuron death	15	3.1	1.37E-02
action potential	8	1.7	2.65E-02
long-term synaptic potentiation	5	1.0	4.96E-02
**Membrane transport activity**	138	33.57	
ion transport	68	14.2	9.28E-06
regulation of transport	65	13.6	3.28E-03
transmembrane transport	60	12.5	2.82E-05
vesicle-mediated transport	49	10.2	2.14E-02
ion transmembrane transport	42	8.8	6.10E-04
regulation of transmembrane transport	17	3.5	4.19E-02
hormone transport	16	3.3	2.22E-02
regulation of peptide transport	14	2.9	2.71E-02
potassium ion transmembrane transport	11	2.3	1.45E-02
negative regulation of transmembrane receptor protein serine/threonine kinase signaling pathway	8	1.7	3.00E-02
positive regulation of transporter activity	6	1.3	3.95E-02
regulation of membrane repolarization	5	1.0	5.38E-03
**Homeostasis**	63	15.32	
chemical homeostasis	42	8.8	1.91E-03
cellular chemical homeostasis	27	5.6	1.49E-02
hemostasis	24	5.0	2.22E-02
regulation of ion homeostasis	15	3.1	4.98E-04

Based on Gene Ontology (DAVID v6.8, Gene_Ontology: GOTERM_BP_4, functional annotation chart, Count: 2, $p < 0.05$)

Table S12. Human diseases corresponding to genes found differentially methylated in sperm cells of F2 offspring of females rats exposed to persistent organic pollutants.

Disease category	Count (total = 359)	%	P value
**METABOLIC**	196	40.9	5.16E-08
Body height	31	6.5	5.51E-04
Triglycerides	28	5.8	7.99E-05
Body mass index	26	5.4	1.72E-03
Body weight	21	4.4	1.00E-02
Cholesterol	20	4.2	2.83E-02
Diabetes mellitus type 2	11	2.3	2.59E-02
Diabetes type 1	11	2.3	3.70E-02
Body fat distribution	8	1.7	2.38E-02
Diabetes mellitus, type 1	5	1.0	1.67E-02
**CARDIOVASCULAR**	161	33.6	1.57E-06
hypertension	32	6.7	6.99E-03
Blood pressure	22	4.6	1.74E-02
Coronary disease	15	3.1	6.61E-03
Atherosclerosis, cardiovascular diseases	4	0.8	6.72E-04
Cardiomyopathy dilated	4	0.8	1.45E-02
**CHEMICAL DEPENDENCY**	160	33.4	1.53E-11
Tobacco use disorder	135	28.2	1.06E-09
Alcoholism	26	5.4	3.88E-03
smoking cessation	10	2.1	2.60E-02
**NEUROLOGICAL**	109	22.8	2.72E-04
Alzheimer's disease	43	9.0	1.59E-03
Stroke	27	5.6	4.37E-03
hypothyroidism	4	0.8	3.74E-02
Brain ischemia, coronary artery disease, intracranial arteriosclerosis|myocardial infarction, stroke	3	0.6	1.45E-02
Parietal lobe	3	0.6	2.40E-02
Sleep-related	8	1.7	1.26E-03
**IMMUNE**	103	21.5	8.55E-03
Asthma	22	4.6	1.66E-02
Hodgkin's disease	5	1.0	9.96E-03
myeloid leukemia	4	0.8	3.74E-02
Arthritis, psoriatic	3	0.6	2.95E-02
**PSYCHIATRIC**	81	16.9	1.86E-04
Bipolar disorder	22	4.6	4.52E-03
several psychiatric disorders	15	3.1	3.83E-02
Neuropsychological tests	5	1.0	3.02E-02
**DEVELOPMENTAL**	62	12.9	1.19E-03
Hip	20	4.2	5.51E-03
Bone density	13	2.7	2.13E-02
Prenatal exposure delayed effects	4	0.8	1.06E-02
Sudden infant death	4	0.8	3.39E-02
Eclampsia, pre-eclampsia	3	0.6	4.39E-03
**RENAL**	61	12.7	4.60E-04
Chronic renal failure, kidney failure, chronic	37	7.7	1.70E-02
**AUTO-IMMUNE**	20	5.5	1.25E-02
Lupus erythematosus, systemic	16	3.3	3.80E-02

Based on Gene Ontology (DAVID v6.8, Gene_Ontology: GOTERM_BP_4, functional annotation chart, Count: 2, $p < 0.05$)

Table S13. Differentially methylated genes conserved in sperm cells of both F1 and F2 progeny of female rats exposed to persistent organic pollutants.

Function	Count (total = 81)	%	P value
**Metabolic pathways**	46	56.79	
phosphate compound metabolism	30	30.9	2.29E-04
positive regulation	29	29.9	1.17E-02
positive regulation of cellular metabolic processes	25	25.8	1.15E-02
positive regulation of macromolecule processes	22	22.7	4.65E-02
positive regulation of protein metabolism	14	14.4	4.79E-02
organophosphate metabolism	13	13.4	6.86E-03
**Lipid-related**			
cellular lipid metabolism	12	12.4	1.06E-02
phospholipid metabolism	7	7.2	1.09E-02
glycerolipid metabolism	6	6.2	4.11E-02
**Signal transduction**	18	22.2	
positive regulation of response to stimulus	18	18.6	3.66E-02
regulation of cell death	16	16.5	9.75E-03
regulation of response to stress	14	14.4	3.71E-02
response to growth factor	10	10.3	4.65E-02
**Immune system and inflammatory response**	31	38.27	
regulation of immune response	12	12.4	1.34E-02
regulation of host defenses	10	10.3	1.79E-02
inflammatory response	10	10.3	3.72E-03
activation of immune response	9	9.3	5.69E-03
platelet activation	5	5.2	3.91E-02
complement activation/humoral immune response	3	3.1	9.85E-03

Based on Gene Ontology (DAVID v6.8, Gene_Ontology: GOTERM_BP_4, functional annotation chart, Count: 2, $p < 0.05$)

Table S14. Human diseases corresponding to genes found methylated differentially in sperm cells of both F1 and F2 progeny of female rats exposed to persistent organic pollutants

Disease category	Count (total = 77)	%	P value
**METABOLIC**	43	44.3	6.64E-03
Body height	8	8.2	4.94E-02
plasma HDL cholesterol (HDL-C) levels	6	6.2	1.53E-02
Diabetes mellitus type 2	5	5.2	2.05E-02
**CHEMICAL DEPENDENCY**	38	39.2	1.04E-04
Tobacco use disorder	34	35.1	8.70E-05
Alcoholism	8	8.2	2.92E-02
**CARDIOVASCULAR**	37	38.1	5.94E-03
**INFECTIOUS**	22	22.7	1.05E-02
Lupus erythematosus, systemic	5	5.2	1.35E-02
natural antibodies	3	3.1	1.48E-03
**RENAL**	16	16.5	1.63E-02
Chronic renal failure, kidney failure	12	12.4	1.59E-02
kidney aging	4	4.1	2.03E-02
**DEVELOPMENTAL**	16	16.5	2.62E-02
Alzheimer's disease	12	12.4	2.20E-02
Sleep-related	3	3.1	4.82E-02

Based on Gene Ontology (DAVID v6.8, Gene_Ontology: GOTERM_BP_4, functional annotation chart, Count: 2, p < 0.05)

Table S15. Biological processes associated with genes found methylated differentially in sperm cells of F1, F2 and F3 progeny of female rats exposed to persistent organic pollutants

Category	Count (total = 36)	%	P value
**Metabolic pathways**	5	13.8	
regulation of lipid metabolism	4	9.1	2.48E-02
neutral lipid biosynthesis	3	6.8	1.49E-02
regulation of lipid biosynthesis	3	6.8	3.38E-02

Based on Gene Ontology (DAVID v6.8, Gene_Ontology: GOTERM_BP_4, functional annotation chart, Count: 2, p < 0.05)

Maurice *et al.* Supplementary data, Table S16

Table S16. Human diseases corresponding to genes found differentially methylated in sperm cells of F1, F2 and F3 progeny of female rats exposed to persistent organic pollutants

Disease category	Count	List Total	%	P value
**METABOLIC**	22	44	50.0	6.22E-03
Triglycerides	5	44	11.4	2.40E-02
plasma HDL cholesterol (HDL-C) levels	4	44	9.1	2.50E-02
Diabetes mellitus type 1	3	44	6.8	3.07E-02
**CARDIOVASCULAR**	37	77	38.1	5.94E-03
**INFECTIOUS**	22	77	22.7	1.05E-02
**DEVELOPMENTAL**	16	77	16.5	2.62E-02
skeletal system development	3	44	6.8	3.39E-02
**RENAL**	16	77	16.5	1.63E-02
Chronic renal failure, kidney failure	7	44	15.9	2.48E-02
**AGING**	7	44	15.9	3.03E-02
kidney aging	4	44	9.1	1.99E-03

Based on Gene Ontology (DAVID v6.8, Gene_Ontology: GOTERM_BP_4, functional annotation chart, Count: 2, p < 0.05)

Table S17. Genes with brain-specific expression found differentially methylated in sperm cells of descendants of female Sprague Dawley rats exposed to POPs

Gene Symbol	Name	Generation	Methylation status	Location
Bai2	brain-specific angiogenesis inhibitor 2	F1	hyper	intron
Dbx1	developing brain homeobox 1	F1	hyper	intron
Bai3	brain-specific angiogenesis inhibitor 3	F2	hyper	intron
Stx1a	syntaxin 1A (brain)	F3	hyper	intron + Exon
Gbx1	gastrulation brain homeobox 1	F3	hypo	exon
Gbx2	gastrulation brain homeobox 2	F3	hypo	intron

Table S18: The quality of sequenced reads for each sample (on library per sample).

Sample	Generation	Group	Number of paired reads	Flagged as poor quality reads		GC (%)	
				R1	R2	R1	R2
JQ1	F1	Control	13524207	0	0	36	35
JQ2	F1	Control	15611177	0	0	36	35
JQ3	F1	Control	15169004	0	0	36	35
JQ4	F1	Control	13393315	0	0	35	34
JQ5	F1	Control	13916208	0	0	36	34
JQ6	F1	Control	13618851	0	0	35	34
JQ7	F1	Case	16358142	0	0	36	35
JQ8	F1	Case	16308465	0	0	36	35
JQ9	F1	Case	15991423	0	0	35	34
JQ10	F1	Case	13313672	0	0	36	35
JQ11	F1	Case	14042897	0	0	36	35
JQ12	F1	Case	12963975	0	0	35	34
F2A	F2	Control	13219848	0	0	37	37
F2B	F2	Control	16574489	0	0	37	37
F2C	F2	Control	14202003	0	0	36	36
F2D	F2	Control	12790005	0	0	36	36
F2E	F2	Control	14276880	0	0	36	36
F2F	F2	Control	14238800	0	0	37	37
F2G	F2	Case	11844150	0	0	38	39
F2H	F2	Case	11966774	0	0	36	36
F2I	F2	Case	17655677	0	0	37	37
F2J	F2	Case	12230558	0	0	36	36
F2K	F2	Case	14706563	0	0	36	36
F2L	F2	Case	14729885	0	0	37	37
F3A	F3	Control	13999362	0	0	36	35
F3B	F3	Control	13635334	0	0	35	34
F3C	F3	Control	12759812	0	0	35	34
F3D	F3	Control	12759958	0	0	35	34
F3E	F3	Control	12341056	0	0	35	34
F3F	F3	Control	13264039	0	0	36	35
F3G	F3	Case	13487337	0	0	36	35
F3H	F3	Case	15194637	0	0	36	36
F3I	F3	Case	14572998	0	0	35	35
F3J	F3	Case	13431040	0	0	36	35
F3K	F3	Case	14236308	0	0	35	35
F3L	F3	Case	13399654	0	0	35	35

Table S19: The quality of alignment for each sample (one library per sample).

Sample	Generation	Group	Mapped (%)	Mapped and properly paired (%)
JQ1	1	Control	73.40	70
JQ2	1	Control	73.06	69
JQ3	1	Control	69.83	66
JQ4	1	Control	70.45	67
JQ5	1	Control	67.19	63
JQ6	1	Control	63.44	59
JQ7	1	Case	67.86	64
JQ8	1	Case	66.00	62
JQ9	1	Case	68.02	64
JQ10	1	Case	72.10	69
JQ11	1	Case	71.00	67
JQ12	1	Case	69.86	66
F2A	2	Control	70.79	67
F2B	2	Control	72.00	68
F2C	2	Control	76.05	73
F2D	2	Control	75.14	72
F2E	2	Control	70.54	67
F2F	2	Control	65.13	61
F2G	2	Case	62.27	57
F2H	2	Case	72.17	68
F2I	2	Case	75.79	73
F2J	2	Case	70.91	67
F2K	2	Case	68.63	65
F2L	2	Case	75.26	72
F3A	3	Control	74.55	71
F3B	3	Control	71.66	68
F3C	3	Control	77.18	74
F3D	3	Control	72.07	69
F3E	3	Control	73.18	70
F3F	3	Control	74.58	72
F3G	3	Case	72.80	69
F3H	3	Case	72.22	69
F3I	3	Case	71.47	68
F3J	3	Case	70.94	67
F3K	3	Case	69.94	66
F3L	3	Case	66.32	62

Maurice *et al.* Supplementary data, Table S20

Table S20: Statistics related to the coverage of the CpG sites identified in each sample.

Sample	Generation	Group	Number of sites	Mean coverage	Standard deviation coverage	Minimum coverage	Maximum coverage
JQ1	F1	Control	1147142	88.03	131.56	15	16510
JQ2	F1	Control	1252836	75.58	99.06	15	12136
JQ3	F1	Control	1189111	77.67	136.30	15	26319
JQ4	F1	Control	1239409	60.67	81.60	15	10796
JQ5	F1	Control	1240470	59.36	77.53	15	9989
JQ6	F1	Control	1254505	53.47	81.64	15	12870
JQ7	F1	Case	1315533	75.15	147.11	15	29656
JQ8	F1	Case	1237065	86.30	146.71	15	28571
JQ9	F1	Case	1310896	69.41	115.49	15	21973
JQ10	F1	Case	1188421	62.05	142.91	15	26909
JQ11	F1	Case	1174421	70.65	144.18	15	28139
JQ12	F1	Case	1184652	61.34	106.88	15	17218
F2A	F2	Control	1291391	63.43	89.84	15	15528
F2B	F2	Control	1352500	74.18	113.30	15	20625
F2C	F2	Control	1167532	78.43	124.03	15	25099
F2D	F2	Control	1242133	59.59	95.54	15	16439
F2E	F2	Control	1329941	61.26	101.92	15	16658
F2F	F2	Control	1197555	62.94	101.53	15	15890
F2G	F2	Case	1093121	49.86	82.32	15	15415
F2H	F2	Case	900941	62.57	114.80	15	18762
F2I	F2	Case	1272404	92.08	162.26	15	28264
F2J	F2	Case	1221013	59.38	105.53	15	16936
F2K	F2	Case	1287416	66.30	97.79	15	16219
F2L	F2	Case	1228249	83.41	132.53	15	20945
F3A	F3	Control	1417749	55.73	85.37	15	18197
F3B	F3	Control	1337204	54.73	83.33	15	14995
F3C	F3	Control	1390480	49.66	75.93	15	14635
F3D	F3	Control	1471278	45.16	79.12	15	11466
F3E	F3	Control	1291387	47.84	76.87	15	15314
F3F	F3	Control	1322370	58.50	99.01	15	17090
F3G	F3	Case	1381526	52.44	91.56	15	17732
F3H	F3	Case	1415542	54.02	139.17	15	27193
F3I	F3	Case	1459241	50.26	91.23	15	17603
F3J	F3	Case	1434430	46.29	73.10	15	12673
F3K	F3	Case	1545507	42.39	55.81	15	10921
F3L	F3	Case	1340657	44.78	73.24	15	14255
